# Characterization of beta-lactam-resistant *Escherichia coli* from Australian fruit bats indicates anthropogenic origins

**DOI:** 10.1099/mgen.0.000571

**Published:** 2021-05-05

**Authors:** Fiona K. McDougall, Wayne S. J. Boardman, Michelle L. Power

**Affiliations:** ^1^​ Department of Biological Sciences, Macquarie University, NSW 2109, Australia; ^2^​ School of Animal and Veterinary Sciences, University of Adelaide, Adelaide, SA 5371, Australia

**Keywords:** antimicrobial resistance, carbapenem, extended-spectrum beta-lactamases, extra-intestinal pathogenic *E. coli*, One Health, zoonoses

## Abstract

Antimicrobial-resistant *Escherichia coli *, particularly those resistant to critically important antimicrobials, are increasingly reported in wildlife. The dissemination of antimicrobial-resistant bacteria to wildlife indicates the far-reaching impact of selective pressures imposed by humans on bacteria through misuse of antimicrobials. The grey-headed flying fox (GHFF; *Pteropus poliocephalus*), a fruit bat endemic to eastern Australia, commonly inhabits urban environments and encounters human microbial pollution. To determine if GHFF have acquired human-associated bacteria, faecal samples from wild GHFF (*n=*287) and captive GHFF undergoing rehabilitation following illness or injury (*n=*31) were cultured to detect beta-lactam-resistant *E. coli *. Antimicrobial susceptibility testing, PCR and whole genome sequencing were used to determine phenotypic and genotypic antimicrobial resistance profiles, strain type and virulence factor profiles. Overall, 3.8 % of GHFF carried amoxicillin-resistant *E. coli* (wild 3.5 % and captive 6.5 %), with 38.5 % of the 13 GHFF *E. coli* isolates exhibiting multidrug resistance. Carbapenem (*bla*_NDM-5_) and fluoroquinolone resistance were detected in one *E. coli* isolate, and two isolates were resistant to third-generation cephalosporins (*bla*_CTX-M-27_ and *ampC*). Resistance to tetracycline and trimethoprim plus sulfamethoxazole were detected in 69.2% and 30.8 % of isolates respectively. Class 1 integrons, a genetic determinant of resistance, were detected in 38.5 % of isolates. Nine of the GHFF isolates (69.2 %) harboured extraintestinal virulence factors. Phylogenetic analysis placed the 13 GHFF isolates in lineages associated with humans and/or domestic animals. Three isolates were human-associated extraintestinal pathogenic *E. coli* (ST10 O89:H9, ST73 and ST394) and seven isolates belonged to lineages associated with extraintestinal disease in both humans and domestic animals (ST88, ST117, ST131, ST155 complex, ST398 and ST1850). This study provides evidence of anthropogenic multidrug-resistant and pathogenic *E. coli* transmission to wildlife, further demonstrating the necessity for incorporating wildlife surveillance within the One Health approach to managing antimicrobial resistance.

## Data Summary

Paired short-read sequence data for 13 antimicrobial-resistant *Escherichia coli* isolated from grey-headed flying foxes have been uploaded to the NCBI Sequence Read Archive (SRA) under BioProject ID PRJNA606529 (https://www.ncbi.nlm.nih.gov/sra/PRJNA606529). Assembled isolate sequences are available in EnteroBase, according to isolate name (http://enterobase.warwick.ac.uk/species/index/ecoli). Class 1 integron sequences were submitted to GenBank under accession numbers MT241250 to MT241254. An IncX3 plasmid sequence was submitted to GenBank under accession number MT264996 (https://www.ncbi.nlm.nih.gov/genbank/). Individual sample SRA and GenBank accession numbers, and EnteroBase Barcodes are available in Table S2 (available in the online version of this article). GitHub URL links for interactive versions of all GrapeTree cgMLST phylogenetic trees are provided in Table S3 (available in the online version of this article).

**Impact Statement**

The spread of antimicrobial-resistant bacteria to wildlife has implications for the continuing emergence of antimicrobial resistance. We undertook studies targeting beta-lactam-resistant *Escherichia coli* in grey-headed flying foxes (GHFF), a fruit bat species endemic to Australia. We identified *E. coli* exhibiting resistance to multiple antimicrobials including several considered critically important in human and veterinary medicine (carbapenems, cephalosporins and fluoroquinolones). The majority of resistant *E. coli* were also characteristic of extraintestinal pathogenic *E. coli *, a type of *E. coli* that can cause urinary tract and blood infections in people. Phylogenetic analysis showed the GHFF *E. coli* isolates to be closely related to isolates associated with humans and/or domestic animals. These findings indicate GHFF have acquired antimicrobial-resistant and pathogenic *E. coli* from humans and domestic animals. Further studies are needed to determine if these *E. coli* pose a zoonotic risk for people and if they impact GHFF health.

## Introduction

Global dissemination of multidrug-resistant (MDR) pathogenic *Escherichia coli* to humans, domestic animals and wildlife is of significant concern [1, 2]. In particular, dissemination of *E. coli* strains exhibiting resistance to critically important antimicrobials (CIAs), including third- to fifth-generation cephalosporins, fluoroquinolones, carbapenems and colistin, is of most concern [3, 4]. The dissemination of multidrug resistance is greatly facilitated via the association of antimicrobial resistance genes with mobile genetic elements (MGEs), such as plasmids and transposons [5]. MGEs enable the horizontal transfer of antimicrobial resistance genes (ARGs) between diverse species of bacteria [5]. Integrons, via their association with transposons and plasmids, have played a significant role in the emergence and spread of antimicrobial resistance (AMR) [6]. Class 1 integrons are capable of capturing and expressing diverse ARGs, mediated by the integrase gene (*intl1*) and a promoter (*Pc*), and typically carry a 3′-conserved segment (*qacE∆1-sul1*) [7]. The clinical class 1 integron is common in Gram-negative bacteria, including MDR pathogenic *E. coli* [6].

The pathogenicity of *E. coli* strains is largely determined by the carriage of virulence factors (VFs) with specific combinations of VFs defining pathogenic types (or pathotypes) [8]. Pathotypes are divided into those causing intestinal disease, referred to as intestinal pathogenic *E. coli* (IPEC), and those causing extraintestinal disease, referred to as extraintestinal pathogenic *E. coli* (ExPEC) [8]. ExPEC are subtyped according to infection site and host, such as uropathogenic *E. coli* (UPEC) in urinary tract infections (UTIs), sepsis-associated *E. coli* (SEPEC), neonatal meningitis *E. coli* (NMEC) and avian pathogenic *E. coli* (APEC) [9]. Core housekeeping genes are used to distinguish *E. coli* sequence types (STs), most commonly using the Achtman seven-gene multilocus sequence typing (MLST) scheme [10]. Specific STs such as ST69, ST73, ST95, ST131 and ST393 represent clonal groups of pathogenic *E. coli* [11], which are often associated with MDR UTI and sepsis [12–14].

MDR *E. coli *, including human pathogenic strains and clonal groups, are increasingly being detected in wildlife species around the world [15–18]. Of significant concern are the growing reports of resistance to CIAs in *E. coli* from wildlife, particularly extended-spectrum beta-lactamase- (ESBL) [19] and carbapenemase-producing *E. coli* [20], conferring resistance to third-generation cephalosporins and carbapenems respectively. An anthropogenic origin of AMR in wildlife is further supported by the higher prevalence of AMR in wildlife species in close proximity to anthropogenic activity [21]. Wildlife species in captivity typically exhibit higher levels of AMR compared to their wild counterparts [22, 23], as do wildlife living or feeding in urban environments, such as silver gulls (*Chroicocephalus novaehollandiae*) [20], house crows (*Corvus splendens*) [16], red foxes (*Vulpes vulpes*) [24] and rats (*Rattus norvegicus* and *R. rattus*) [15]. Wild birds, particularly migratory species, may play a significant role in the long-distance dissemination of CIA-resistant *E. coli* [25].

Bats are the only mammals to have achieved powered flight [26], and like birds, they are highly mobile and capable of flying long distances [27, 28]. There are over 1300 species of bats belonging to the order Chiroptera [29], which is divided into two suborders: Yangochiroptera (includes the majority of microbat species) and Yinpterochiroptera (includes fruit bats and flying foxes) [30]. *E. coli* is reported as a common component of the intestinal microbiome in diverse species of microbats [31, 32] and fruit bats [32–36]. MDR and ESBL-producing *E. coli* have been detected in microbat species in Peru [37], Poland [38] and Portugal [39], and ExPEC-associated VFs were detected in antimicrobial-resistant *E. coli* from microbats in Brazil [31] and Portugal [39]. Of four studies investigating AMR in *E. coli* from fruit bat species, MDR and ESBL-producing *E. coli* were isolated from Franquet’s epauletted fruit bats (*Epomops franqueti*), Woermann’s fruit bats (*Megaloglossus woermanni*) [40] and straw coloured fruit bats (*Eidolon helvum*) (A. O. Oluduro, pers. comm.) in Africa [41]. The detection of AMR in fruit bats dates to as early as 1985, with trimethoprim, sulfamethoxazole and first-generation cephalosporin-resistant *E. coli* found in fruit bats (*Cynopterus* sp.) in Indonesia [42]. In contrast, *E. coli* isolates from five fruit bat species in the Republic of Congo did not exhibit acquired AMR, but multiple isolates were carrying ExPEC-associated VFs and 38.5 % of *E. coli* isolates were assigned to human- and domestic animal-associated STs (ST69, ST101, ST127, ST131 and ST372) [33]. To date, no studies have performed whole genome sequencing (WGS) or phylogenetic analysis on *E. coli* isolates from either microbats or fruit bats.

Grey-headed flying foxes (GHFF; *Pteropus poliocephalus*) are a large fruit bat species endemic to Australia, with a broad geographical range extending across four States in eastern Australia. GHFF colonies typically comprise several thousand individuals and may contain upwards of 50 000 flying foxes. Over recent decades, the number of colonies in urban environments, and the number of GHFF occupying urban colonies, has been increasing [43, 44], thus creating greater connectivity between people and GHFF in urban Australia.

Beta-lactam-resistant *E. coli* have previously been reported in several non-Australian fruit bat species [40–42], but no studies have examined the carriage of beta-lactam-resistant *E. coli* in Australian fruit bats. However, ARGs to narrow-spectrum penicillins, trimethoprim and aminoglycosides were detected in class 1 integrons in faecal DNA from GHFF, indicating carriage of resistance determinants by intestinal bacteria [45]. This study investigated the occurrence and genetic mechanisms of beta-lactam and multidrug resistance in *E. coli* isolated from wild GHFF in urban colonies and captive GHFF undergoing rehabilitation following illness or injury. *E. coli* exhibiting AMR were assessed for human-associated STs and virulence determinants associated with specific pathotypes of *E. coli *.

## Methods

### Faecal sample collection

A total of 318 faecal samples from wild GHFF (*n*=287) and captive GHFF (*n*=31) were used in this study. Captive GHFF were injured or sick flying foxes (from wild colonies) undergoing rehabilitation. Samples representing wild GHFF were obtained from three locations: Sydney, New South Wales (NSW) (*n*=61); Lake Macquarie, NSW (*n*=121); and Adelaide, South Australia (SA) (*n*=104). Captive GHFF samples were obtained via two wildlife rehabilitation organizations: Fauna Rescue of South Australia (SA) (*n*=19) and Wildlife Information, Rescue and Education Service NSW (WIRES) (*n*=12). The captive GHFF in SA were recovering from heat stress and did not receive antimicrobial therapy and veterinary treatment records were unavailable for the NSW captive GHFF.

Faecal samples were acquired either directly from individual GHFF or opportunistically under roosting flying foxes. Using a FecalSwab system (COPAN), discrete faecal samples were collected from plastic drop sheets placed under roosting flying foxes (*n*=194), via a rectal swab (*n*=94; Adelaide) or collected aseptically from the intestine at necropsy (*n*=30; Adelaide and Sydney). Euthanized and freshly deceased GHFF were frozen at −20 °C and thawed for necropsy and sampling within 4–8 weeks. FecalSwab samples were stored at 4 °C and cultured within 72 h of collection.

### Detection of beta-lactam-resistant *E. coli* in faecal samples

Faecal samples were screened for the presence of beta-lactam-resistant *E. coli* (specifically, resistance to penicillins and third-generation cephalosporins) by inoculating FecalSwab media (0.2 ml) into 5 ml of Luria-Bertani (LB) broth (Difco Laboratories) containing 10 mg l^−1^ amoxicillin (a penicillin) (Sigma), and incubated overnight at 37 °C. The LB broth was then inoculated onto Chromocult Coliform Agar (Merck Millipore) supplemented with 10 mg l^−1^ amoxicillin (Sigma) or 32 mg l^−1^ cefoperazone (a third-generation cephalosporin) (Oxoid) and incubated overnight at 37 °C. Dark blue or purple colonies were deemed to be *E. coli *.

### Antimicrobial susceptibility testing of *E. coli* isolates

Isolates exhibiting beta-lactam resistance underwent further antimicrobial susceptibility testing (AST) against a panel of 19 antibiotics comprising 12 antimicrobial categories using disc diffusion and minimal inhibitory concentration (MIC) methods. Disc diffusion AST was performed according to the European Committee on Antimicrobial Susceptibility Testing (EUCAST) with 18 antibiotics comprising 11 antimicrobial categories [46] (Table S1). Isolates were evaluated as susceptible or resistant using EUCAST breakpoint criteria (v 9.0 available at http://www.eucast.org/clinical_breakpoints/). Where EUCAST breakpoints were unavailable, susceptibility was determined using the Clinical and Laboratory Standards Institute (CSLI) breakpoint criteria (CLSI M100 ED29 : 2019 available at https://clsi.org/standards/products/free-resources/access-our-free-resources/).

A negative control, *E. coli* isolate FF1170 (Enterobase Barcode ESC_JA9915AA) was sourced from a GHFF faecal sample cultured on non-supplemented Chromocult Coliform Agar (Merck Millipore). Isolate FF1170 carried no ARGs (previously determined by WGS) and was phenotypically susceptible to all 19 antibiotics used for AST. FF1170 was included as a quality control and to assist evaluating zone diameters where no EUCAST or CLSI breakpoint criteria existed. In the absence of breakpoint data, growth up to the edge of the disc was evaluated as resistant, and intermediate resistance was reported where inhibition zone diameters were smaller than the negative control isolate FF1170, but growth was not up to the edge of the disc. Multidrug resistance was defined as acquired resistance to at least one agent in three or more antimicrobial categories [47].

MIC methods were used for AST of colistin and imipenem. MIC determination of colistin was performed according to EUCAST guidelines and the ISO-standard broth microdilution method (20776-1), using cation-adjusted BBL Mueller-Hinton II Broth (Becton Dickinson) and colistin sulphate salt ≥15 000 U mg^−1^ (Sigma). Isolates exhibiting carbapenem resistance in EUCAST disc diffusion AST (imipenem IPM10 and meropenem MEM10) were further tested to determine the MIC of imipenem using M.I.C. Evaluator strips (Oxoid).

### Screening for class 1 integrons in *E. coli* isolates

Isolates were grown in 10 ml of LB broth (Difco Laboratories) at 37 °C overnight with shaking (150 r.p.m.) and DNA extracted using the ISOLATE II Genomic DNA Kit (Bioline). DNAs were then screened for the presence of the class 1 integron integrase gene (*intI1*) using primers HS463a and HS464, and *intI1* positives were then amplified using primers HS458 and HS459 (target the conserved *attl1* and 3′ *qacE∆1* region of the class 1 integron) [45]. *IntI1*-positive isolates which failed to amplify gene cassette arrays using primers HS458 and HS459 were amplified using primers HS458 and JL-D2 [48] using cycling conditions of 94 °C for 3 min; 35 cycles 94 °C for 30 s, 55 °C for 30 s and 72 °C for 1 min 30 s; and 72 °C for 5 min. JL-D2 targets the IS*26* transposase, an alternativee 3′ sequence to the *qacE∆1*-conserved segment found in class 1 integrons [48]. The HS458/HS459 and HS458/JL-D2 PCRs both amplify the entire class 1 integron gene cassette array.

PCR amplicons were purified for sequencing using the MinElute PCR Purification Kit (Qiagen). Sequencing was performed at the Ramaciotti Centre for Genomics (Sydney, NSW, Australia) using Big Dye Terminator chemistry version 3.1 and ABI 3730/3730xl Capillary Sequencers (Applied Biosystems). Sequences were manually checked for quality, assembled using Geneious R11 software (Biomatters) and analysed for the presence of AMR genes using Integrall (http://integrall.bio.ua.pt/?search#). Annotation was performed manually in Geneious using BLASTn (https://blast.ncbi.nlm.nih.gov/Blast.cgi). Class 1 integron sequences were submitted to GenBank (Table S2).

### Whole genome sequencing of *E. coli* isolates

Isolates were grown in 5 ml LB broth culture at 35 °C overnight with shaking (150 r.p.m.) and DNA extracted as above. Genomic DNA concentrations were determined using a Qubit dsDNA BR assay kit (Invitrogen). Libraries were prepared using Nextera XT DNA or Nextera DNA Flex kits (Illumina) according to the manufacturer’s instructions. WGS was performed on an Illumina MiSeq system using the MiSeq reagent kit v2 (2×150 bp paired-end reads) or MiSeq reagent kit v3 (2×300 bp paired-end reads). Raw sequence reads were assembled as *de novo* genome sequences using SPAdes Assembler 3.13.0 [49] in Geneious Prime 2020.2.1 (Biomatters). Raw sequence reads for all antimicrobial-resistant *E. coli* isolates were uploaded to the NCBI Sequence Read Archive (SRA). Individual SRA accession numbers and EnteroBase Barcodes are listed in Table S1.

ResFinder 4.0 (available at https://cge.cbs.dtu.dk/services) was used to identify acquired AMR genes and point mutations in WGS SPAdes assemblies for each isolate, with search parameters set at a 90 % threshold for identity and 60 % minimum length [50]. Isolates uploaded in EnteroBase were assigned to an *E. coli* phylogroup using ClermonTyping [51], ST using the Achtman seven gene MLST scheme, predicted serotype (O:H) and *fimH* type using fimTyper [52] (http://enterobase.warwick.ac.uk/species/index/ecoli) [53].

WGS SPAdes assemblies for all isolates were screened for VFs associated with IPEC and ExPEC using VirulenceFinder 2.0, with search parameters set at a 90 % threshold for identity and 60 % minimum length (available at https://cge.cbs.dtu.dk/services) [54] and ABRicate VFDB (https://github.com/tseemann/abricate) [55], with search parameters set at a 80 % threshold for identity and 80 % DNA coverage in Galaxy Australia (available at https://usegalaxy.org.au/). Isolates were assessed for the presence of 27 ExPEC-associated VFs: adhesins (*afa/dra*, *fimH*, *iha*, *papA/papC*, *sfa*/*foc*, *tsh*), invasins (*gimB*, *ibeA*), iron acquisition (*fyuA/irp/ybt*, *ireA*, *iroN*, *iutA/iucA*, *sitA*), protectins (*iss*, *neuC, traT*), toxins (*astA*, *clb*, *cnf1*, *hly*, *sat*, *usp, vat*), miscellaneous (*ompT*, *pic*, *malX*) and capsule (*kpsM* II) [9, 56, 57]. Isolates were also assessed for the presence of additional VFs, including bacteriocins (colicins and microcins), *chuA, lpfA* and *senB*.

Isolate pathotype was designated according to carriage of ExPEC-associated and additional VFs. Of the 27 ExPEC-associated VFs, five were considered key VFs (*afa/dra*, *iutA*, *kpsM* II, *papA/papC*, *sfa*/*foc*) [56]. The presence of two or more of these five key ExPEC VFs was used define isolates as ExPEC [56]. Isolates that carried fewer than two of the five key ExPEC VFs were defined as ‘ExPEC-potential’, ‘ExPEC-like’ or ‘low pathogenicity’ as follows: ‘ExPEC-potential’ if they carried five or more of 27 ExPEC-associated VFs, ‘ExPEC-like’ if they carried fewer than five ExPEC VFs but five or more total VFs (ExPEC-associated and additional VFs), or ‘low pathogenicity’ if they carried fewer than five total VFs.

ST131 isolates were assigned to a clade according to *fimH* type, *gyrA* and *parC* allele types (and associated phenotypic resistance or sensitivity to fluoroquinolones), and the presence or absence of *bla*_CTX-M-15_ [2, 58]. ST131 isolates were also assigned a virotype according to a scheme based on the presence or absence of 11 VFs [13].

Plasmids were detected using PlasmidFinder 2.1, with search parameters set at a 95 % threshold for identity and 60 % minimum length for the *Enterobacteriaceae* database [59] (available at https://cge.cbs.dtu.dk/services). The *bla*_NDM-5_ IncX3, *bla*_NDM-7_ IncX3 and *bla*_NDM-1_ plasmids were assembled using Geneious Prime 2020.1.1 1 (Biomatters). IncX3 plasmid annotation was performed using a reference library comprising GenBank accessions (CP032424, MG825368, MG825382, MG825384, MH347484, MH917280) with >99.99 % identity match to the FF993W IncX3 plasmid in a BLASTn search (https://blast.ncbi.nlm.nih.gov/Blast.cgi). The FF993W IncX3 plasmid sequence was submitted to GenBank under accession number MT264996.

### Phylogenetic analysis of *E. coli* isolates

*E. coli* isolates phylogenetically related to GHFF isolates were identified in EnteroBase by searching for isolates with the same ST using the Achtman seven gene MLST scheme (http://enterobase.warwick.ac.uk/species/ecoli/search_strains?query=st_search) [53]. Where fewer than 50 isolates of the same ST were found, the search was expanded to include STs with up to two locus variants. Phylogenetic analysis comparing EnteroBase and GHFF isolates was performed using GrapeTree to construct a rapid neighbour-joining (RapidNJ) minimum spanning tree based on the core-genome MLST (cgMLST) V1 +Hierarchical Clustering (HierCC) V1 scheme from EnteroBase [60]. For trees comprising in excess of 250 isolates, clades containing the GHFF isolate were identified and used to reconstruct refined trees. All resulting trees contained between 17 and 204 isolates. Branch lengths were used to calculate the cgMLST allelic differences between closely related isolates. GitHub URL links for interactive versions of all GrapeTree cgMLST phylogenetic trees are provided in Table S3.

**Fig. 1.**
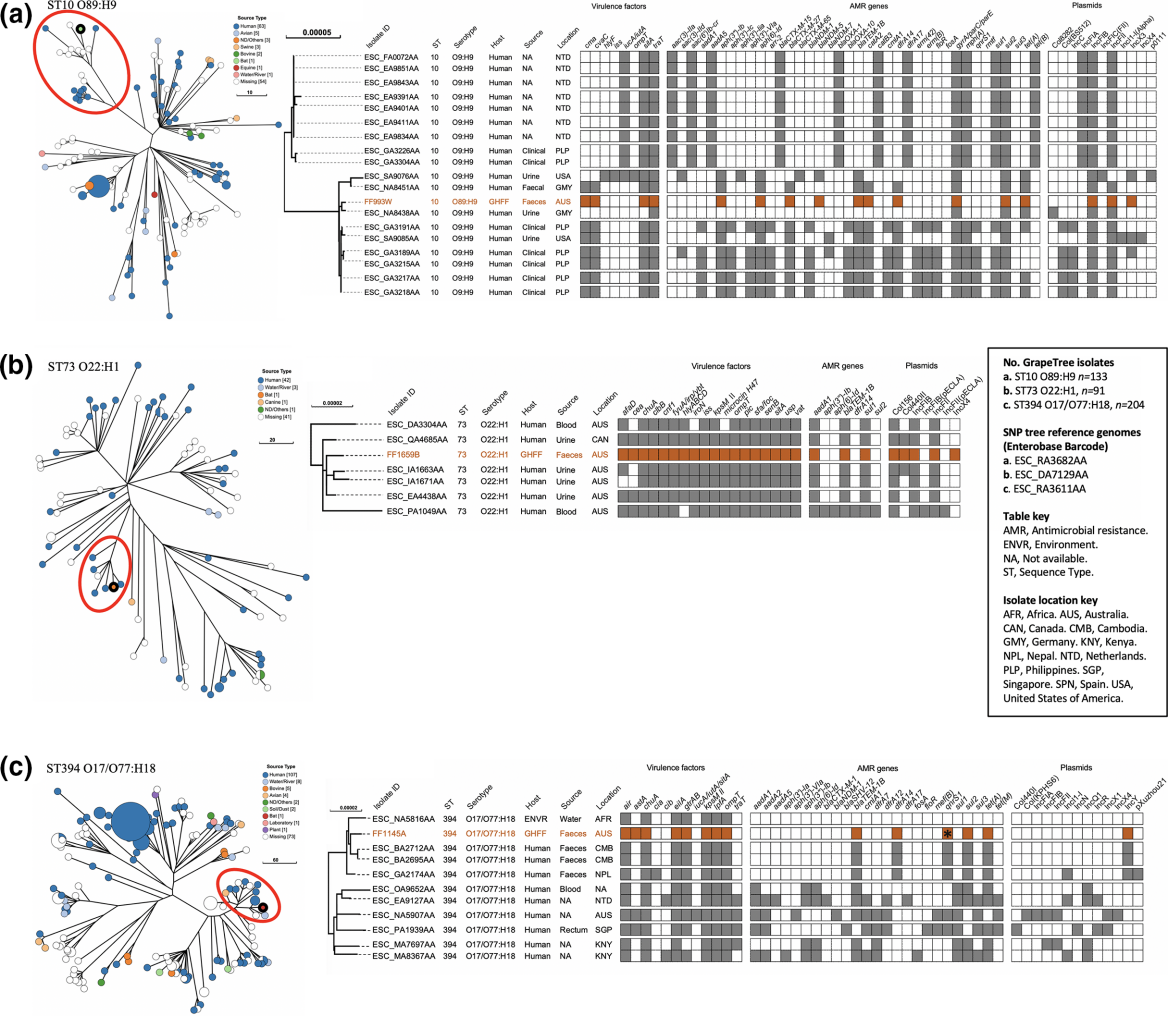
Phylogenetic and metadata analysis of human-associated amoxicillin-resistant extra-intestinal pathogenic *E. coli* isolates from grey-headed flying foxes (GHFF) and closely related isolates identified in Enterobase. Left: GrapeTree phylogeny reconstructed using a rapid neighbour-joining (RapidNJ) minimum spanning tree based on the cgMLST V1+Hierarchical Clustering (HierCC) V1 scheme. GHFF isolates are described as Source Type ‘Bat’ and highlighted with a black circle. Clusters containing GHFF isolates are circled in red. Scale bars indicate the number of cgMLST allelic differences. GitHub URL links for interactive versions of all GrapeTrees are provided in Table S3. Right: core genome SNP analysis and associated metadata tables of GrapeTree clusters containing GHFF isolates. Maximum-likelihood trees were based on RAxML of non-repetitive core SNPs using the EnteroBase SNP Project dendrogram module against a reference genome (removed from SNP tree images for clarity). Isolate ID indicates Enterobase Barcode or GHFF isolate name. Coloured rectangles (orange for GHFF and grey for other host sources) indicate the presence of a specific gene and white squares indicate its absence. Orange text indicates GHFF isolates. Scale bars indicate the number of substitutions per site.** (a**) FF993W, ST10 O89:H9. (**b**) FF1659B, ST73 O22:H1. (**c**) FF1145A, ST394 O17/O77:H18.

**Fig. 2.**
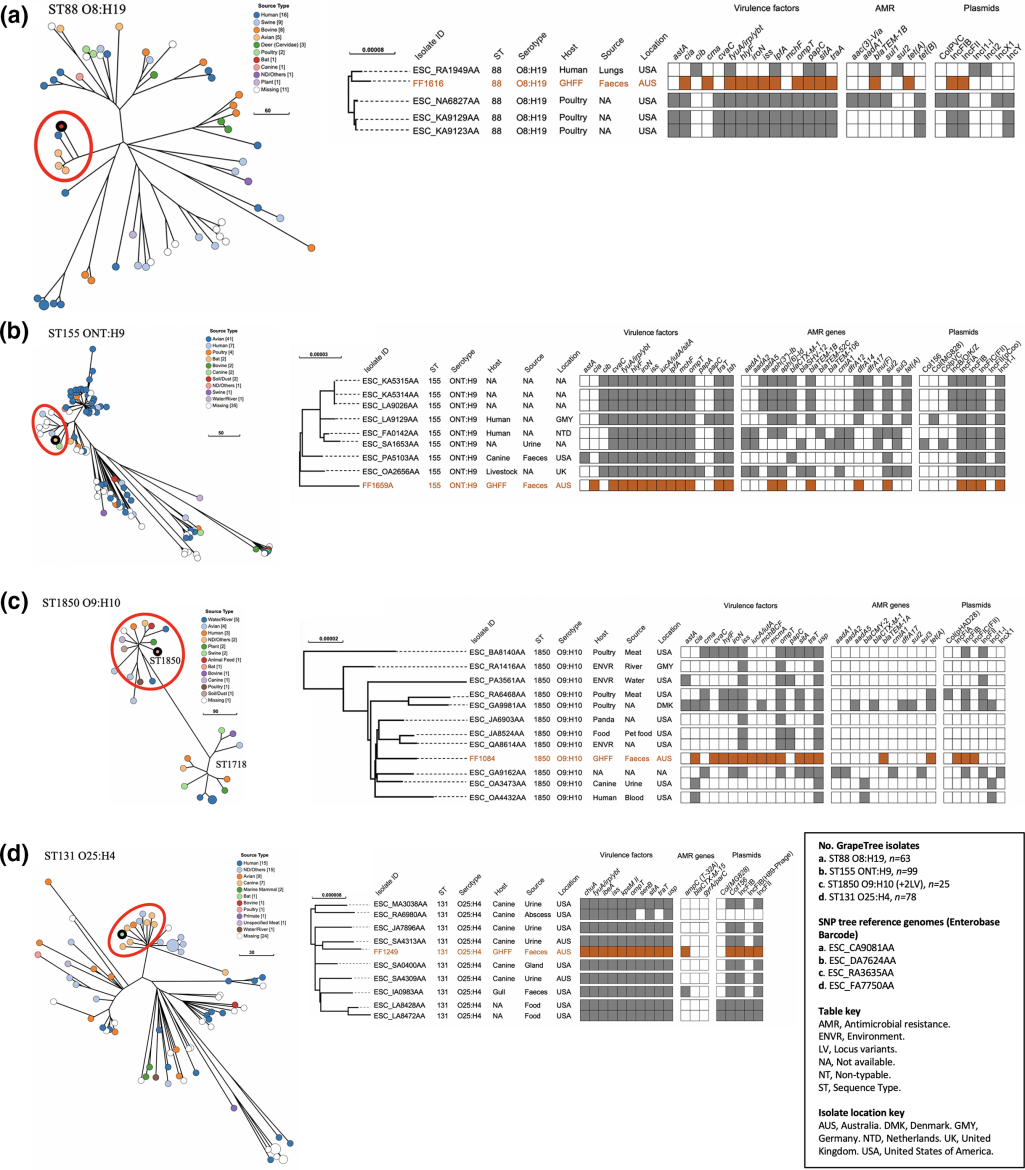
Phylogenetic and metadata analysis of human- and animal-associated amoxicillin-resistant extra-intestinal pathogenic *E. coli* isolates from grey-headed flying foxes (GHFF) and closely related isolates identified in Enterobase. Left: GrapeTree phylogeny reconstructed using a rapid neighbour-joining (RapidNJ) minimum spanning tree based on the cgMLST V1+Hierarchical Clustering (HierCC) V1 scheme. GHFF isolates are described as Source Type ‘Bat’ and highlighted with a black circle. Clusters containing GHFF isolates are circled in red. Scale bars indicate the number of cgMLST allelic differences. GitHub URL links for interactive versions of all GrapeTrees are provided in Table S3. Right: core genome SNP analysis and associated metadata tables of GrapeTree clusters containing GHFF isolates. Maximum-likelihood trees were based on RAxML of non-repetitive core SNPs using the EnteroBase SNP Project dendrogram module against a reference genome (removed from SNP tree images for clarity). Isolate ID indicates Enterobase Barcode or GHFF isolate name. Coloured rectangles (orange for GHFF and grey for other host sources) indicate the presence of a specific gene and white squares indicate its absence. Orange text indicates GHFF isolates. Scale bars indicate the number of substitutions per site.** (a**) FF1616, ST88 O8:H19. (**b**) FF1659A, ST155 ONT:H9. (**c**) FF1084, ST1850 O9:H10. (**d**) FF1249, ST131 O25:H4.

**Fig. 3.**
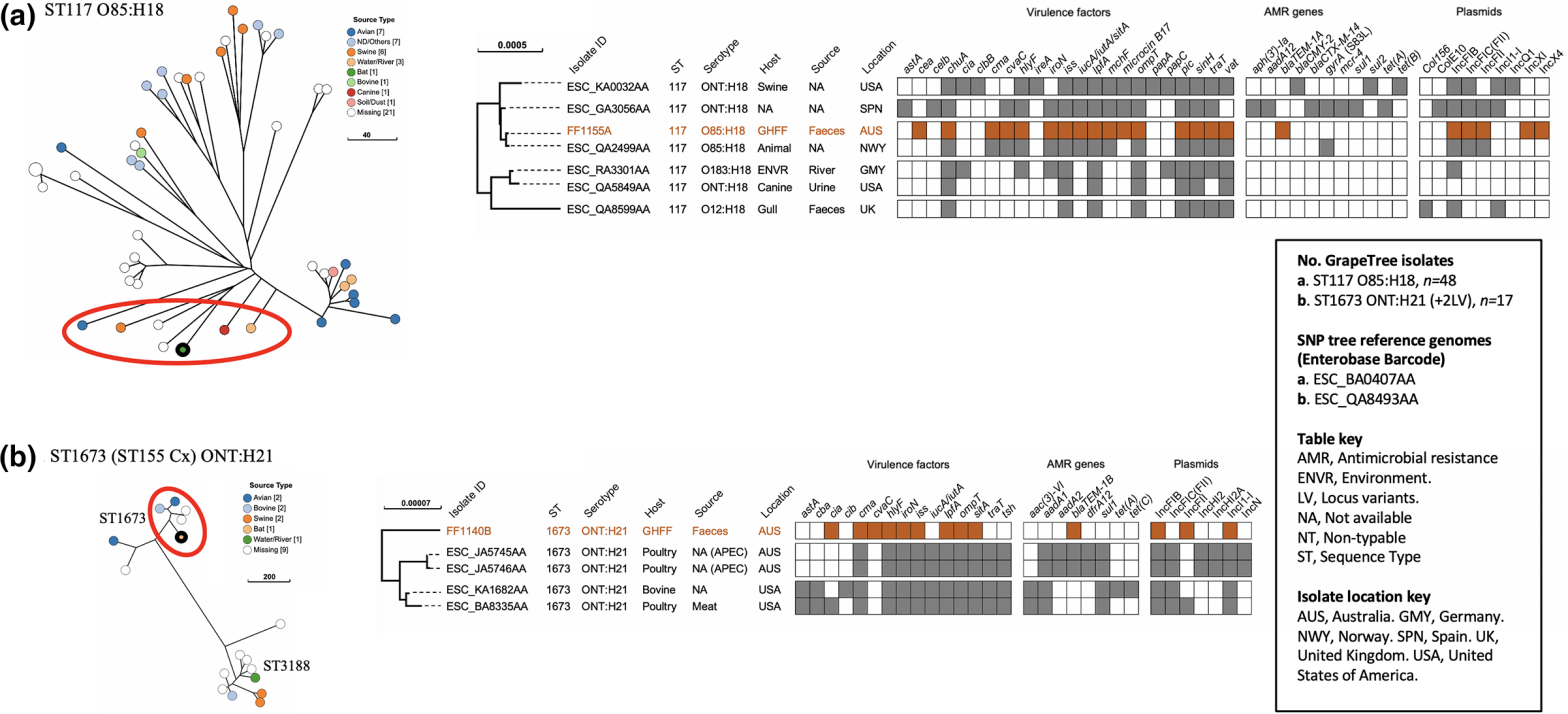
Phylogenetic and metadata analysis of animal-associated amoxicillin-resistant extra-intestinal pathogenic *E. coli* isolates from grey-headed flying foxes (GHFF) and closely related isolates identified in Enterobase. Left: GrapeTree phylogeny reconstructed using a rapid neighbour-joining (RapidNJ) minimum spanning tree based on the cgMLST V1+Hierarchical Clustering (HierCC) V1 scheme. GHFF isolates are described as Source Type ‘Bat’ and highlighted with a black circle. Clusters containing GHFF isolates are circled in red. Scale bars indicate the number of cgMLST allelic differences. GitHub URL links for interactive versions of all GrapeTrees are provided in Table S3. Right: core genome SNP analysis and associated metadata tables of GrapeTree clusters containing GHFF isolates. Maximum-likelihood trees were based on RAxML of non-repetitive core SNPs using the EnteroBase SNP Project dendrogram module against a reference genome (removed from SNP tree images for clarity). Isolate ID indicates Enterobase Barcode or GHFF isolate name. Coloured rectangles (orange for GHFF and grey for other host sources) indicate the presence of a specific gene and white squares indicate its absence. Orange text indicates GHFF isolates. Scale bars indicate the number of substitutions per site.** (a**) FF1155A, ST117 O85:H18. (**b**) FF1140B, ST1673 ONT:H21.

**Fig. 4.**
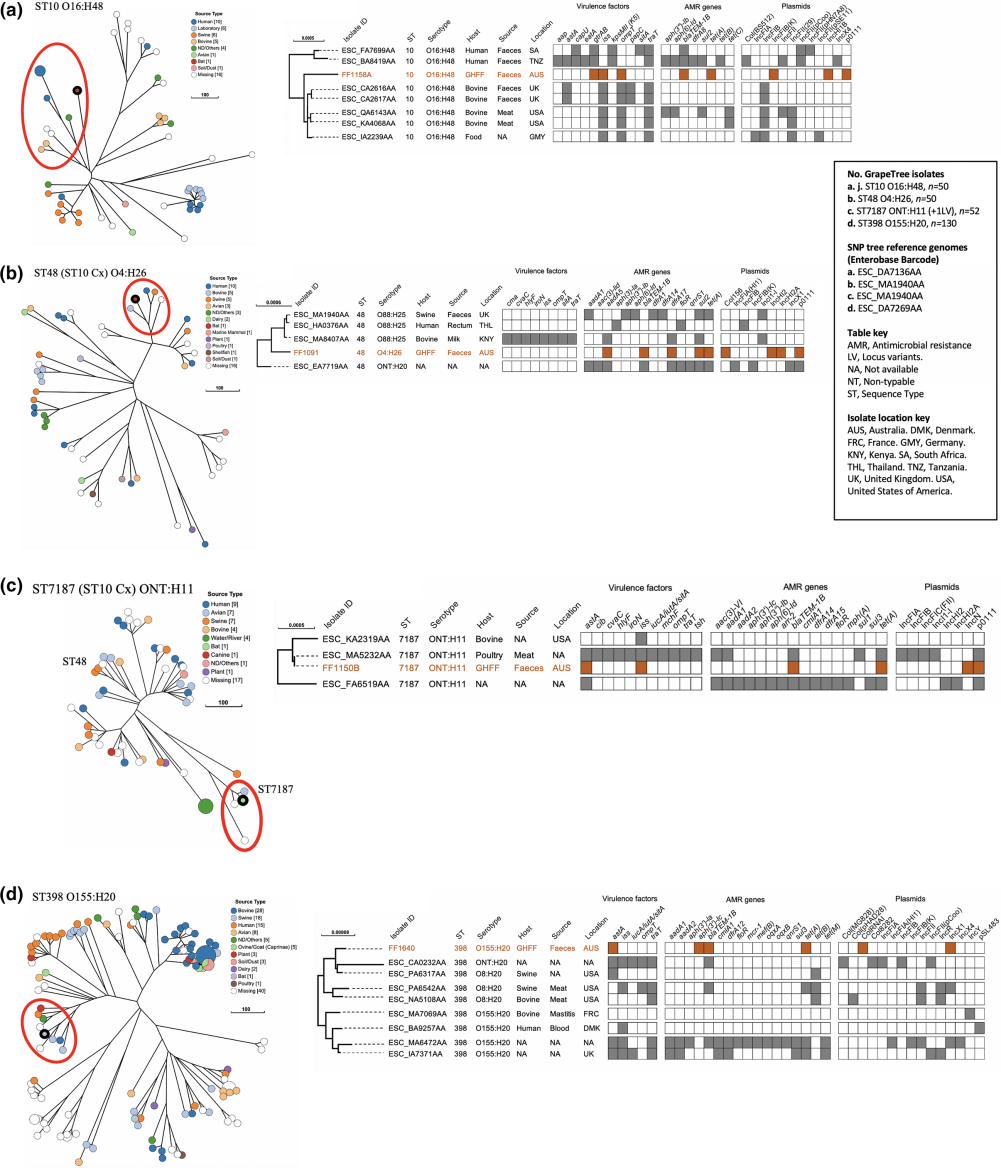
Phylogenetic and metadata analysis of amoxicillin-resistant *E. coli* isolates with low pathogenicity from grey-headed flying foxes (GHFF) and closely related isolates identified in Enterobase. Left: GrapeTree phylogeny reconstructed using a rapid neighbour-joining (RapidNJ) minimum spanning tree based on the cgMLST V1+Hierarchical Clustering (HierCC) V1 scheme. GHFF isolates are described as Source Type ‘Bat’ and highlighted with a black circle. Clusters containing GHFF isolates are circled in red. Scale bars indicate the number of cgMLST allelic differences. GitHub URL links for interactive versions of all GrapeTrees are provided in Table S3. Right: core genome SNP analysis and associated metadata tables of GrapeTree clusters containing GHFF isolates. Maximum-likelihood trees were based on RAxML of non-repetitive core SNPs using the EnteroBase SNP Project dendrogram module against a reference genome (removed from SNP tree images for clarity). Isolate ID indicates Enterobase Barcode or GHFF isolate name. Coloured rectangles (orange for GHFF and grey for other host sources) indicate the presence of a specific gene and white squares indicate its absence. Orange text indicates GHFF isolates. Scale bars indicate the number of substitutions per site.** (a**) 1158A, ST10 O16:H48. (**b**) FF1091, ST48 O4:H26. (**c**) FF1150B, ST7187 ONT:H11. (**d**) FF1640, ST398 O155:H20.

Clusters containing GHFF isolates identified in the GrapeTree phylogenetic analysis were used to construct a maximum-likelihood tree [53] based on RAxML of non-repetitive core SNPs (minimum presence 95 %) using the EnteroBase SNP Project dendrogram module against an appropriate reference genome (Enterobase barcodes for reference genomes are provided in the legends to Figs 1–4) [53]. Metadata and WGS assemblies for SNP cluster isolates were downloaded from EnteroBase [53] and analysed for VFs, AMR genes and plasmids as described for GHFF isolates. Additional metadata was obtained for ST10 O9:H9 isolates from the Philippines (BioProject PRJEB17615) [61] and German isolates ESC_NA8438AA and ESC_NA8451AA (J. B. Hans and the National Reference Laboratory for multidrug-resistant Gram-negative bacteria, Bochum, Germany; pers. comm.), and ST73 isolate ESC_EA4438AA (D. M. Gordon, Australian National University, pers. comm.).

## Results

### Selective culture for beta-lactam-resistant *E. coli* in GHFF faecal samples

Beta-lactam-resistant *E. coli* were isolated from 12 of 318 faecal samples collected from wild and captive GHFF, giving an overall occurrence of 3.8 % (Table 1). The occurrence was 3.5 % (*n=*10/287) in the wild GHFF and 6.5 % (*n=*2/31) in the captive GHFF, and the two captive locations differed considerably (Sydney, 0.0 % and Mylor, 10.5 %) (Table 1). Amoxicillin-resistant *E. coli* (*n*=13) were present in 12 GHFF faecal samples, with two morphologically distinct *E. coli* isolates (determined by colony colour) detected in one faecal sample (FF1659) (Table 1). A single isolate also exhibited resistance to cefoperazone (abundant growth at 32 mg l^−1^) and a second isolate exhibited intermediate resistance to cefoperazone (low growth at 32 mg l^−1^) (Table 2).

**Table 1.** Occurrence of amoxicillin-resistant (AMX-R) *E. coli* detected by location

Location	Wild/captive	No. of faecal samples tested	No. of faecal samples positive for AMX-R *E. coli *	No. AMX-R *E. coli* isolated
SYD	Wild	61	4/61 (6.6 %)	4
LM	Wild	122	3/122 (2.5 %)	4
ADL	Wild	104	3/104 (2.9 %)	3
Total (wild)		287	10/287 (3.5 %)	11
SYD	Captive	12	0/12 (0.0 %)	0
MYL	Captive	19	2/19 (10.5 %)	2
Total (captive)		31	2/31 (6.5 %)	2
Total (wild and captive)		318	12/318 (3.8 %)	13

ADL, Adelaide. AMX-R, Amoxicillin-resistant. LM, Lake Macquarie. MYL, Mylor. SYD, Sydney.

**Table 2.** Phylotyping, sequence typing, serotyping, genotypic and phenotypic resistance profiles and pathotypes of *E. coli* isolates detected in GHFF

Isolate ID	Site	PG	ST	Serotype	*fimH* type	Antimicrobial resistance genes (corresponding EUCAST phenotypic antibiotic resistance)	No. of AM categories	No. of ExPEC VFs (no. of key ExPEC VFs)	No. of additional VFs (total no. of VFs)	Pathotype
FF993W	SYD*	A	10	O89:H9	54	*aph(3'')-Ib+aph(6)-Id* (S), *bla*CTX-M-27 (AMC, CL, KZ, CTX, CFP), *bla*NDM-*5* (IPM, MEM), *bla*TEM-1B (AMX, AMP), *catA2* (C), *dfrA14+sul2* (W, SXT), *parC/parE/gyrA* (NA, CIP), *tet(A)* (TE)	10	3 (0)	2 (5)	ExPEC-like
FF1084	MYL*	A	1850	O9:H10	1113	*bla*TEM-1B (AMX, AMP), *tet(A*) (TE)	2	9 (1)	4 (13)	ExPEC-potential
FF1091	MYL*	A	48 (ST10 Cx)	O4:H26	23	*aadA5 (SH*†*), bla*TEM-1B (AMX, AMP), *dfrA17+sul2* (W, SXT), *tet(A*) (TE)	4	1 (0)	0 (1)	Low pathogenicity
FF1140B	SYD*	B1	1673 (ST155 Cx)	ONT:H21	35	*bla*TEM-1B (AMX, AMP)	1	6 (0)	4 (10)	ExPEC-potential
FF1145A	SYD*	D	394 CGA	O17/O77:H18	30	*bla*TEM-1B (AMX, AMP), *dfrA14+sul2* (W, SXT), *qnrS1* (‡)*, tet(A*) (TE)	3	4 (1)	5 (9)	ExPEC-like
FF1150B	ADL§	A	7187 (ST10 Cx)	ONT:H11	nt	*bla*TEM-1B (AMX, AMP), *tet(A*) (TE)	2	3 (0)	0 (3)	Low pathogenicity
FF1155A	ADL§	G	117	O85:H18	97	*bla*TEM-1A (AMX, AMP), (TE)||	2	10 (1)	8 (18)	ExPEC-potential
FF1158A	ADL§	A	10	O16:H48	86	*bla*TEM-1B (AMX, AMP), *tet(A*) (TE)	2	3 (0)	1 (4)	Low pathogenicity
FF1249	SYD§	B2	131	O25:H4	22	*ampC* (AMX, AMP, AMC, CL, KZ, CT X¶, CFP**)	4	9 (1)	2 (11)	ExPEC-potential
FF1616	LM*	C	88 (ST23 Cx)	O8:H19	27	*bla*TEM-1B (AMX, AMP), *tet(A*) (TE)	2	9 (1)	3 (12)	ExPEC-potential
FF1640	LM*	A	398	O155:H20	54	aph(3')-Ia (‡), blaTEM-1B (AMX, AMP), tet(A) (TE)	2	2 (0)	0 (2)	Low pathogenicity
FF1659A	LM*	B1	155	ONT:H9	366	*aph(3'')-Ib+aph(6)-Id* (S), *bla*TEM-1B (AMX, AMP), *dfrA14+sul2* (W, SXT)	3	10 (1)	4 (14)	ExPEC-potential
FF1659B	LM*	B2	73	O22:H1	9	*aadA1* (SH, S†), *bla*TEM-1B (AMX, AMP)	2	16 (3)	4 (20)	ExPEC

*Isolate obtained from faecal samples collected from plastic drop sheets under GHFF roosts.

†Intermediate resistance to streptomycin (S) or spectinomycin (SH) as determined by inhibition zone diameters < negative control isolate FF1170, but growth not up to the disc.

‡Resistance gene identified in whole genome sequencing, but corresponding phenotypic resistance not exhibited.

§Isolate obtained from faecal samples collected at necropsy from euthanized or freshly deceased wild GHFF.

||Phenotypic resistance present, but antimicrobial resistance gene not identified.

¶Intermediate resistance to cefotaxime (CTX) as determined by EUCAST breakpoint criteria.

**Intermediate resistance to cefoperazone (CFP) as determined by CLSI breakpoint criteria (MIC intermediate resistance=32 mg l^−1^).

ADL, Adelaide; AM, antimicrobial; CGA, clonal group A; Cx, complex; ExPEC, extraintestinal pathogenic *E. coli;* I, intermediate resistance; LM, Lake Macquarie; MYL, Mylor; NT, non-typable; PG, phylogroup; ST, sequence type; SYD, Sydney. VF, virulence factor. Antibiotics: AMC, amoxicillin-clavulanic acid; AMP, ampicillin; AMX, amoxicillin; C, chloramphenicol; CFP, cefoperazone; CIP, ciprofloxacin; CL, cephalexin; CTX, cefotaxime; KZ, cefazolin; IPM, imipenem; MEM, meropenem; NA, naladixic acid; TE, tetracycline; S, streptomycin; SH, spectinomycin; SXT, trimethoprim and sulfamethoxazole; W, trimethoprim.

### Strain typing of GHFF *E. coli* isolates

The 13 amoxicillin-resistant *E. coli* isolates belonged to six phylogroups, with 46.6 % (*n=*6/13) of isolates designated phylogroup A, and the remaining seven isolates distributed between phylogroups B1, B2, C, D and G (Table 2). The 13 GHFF isolates were assigned to 12 different STs (Table 2), of which four isolates belonged to the ST10 complex (ST10, ST48 and ST7187) and two belonged to the ST155 complex (ST155 and ST1673) (Table 2). All 13 isolates were assigned different serotypes, with three designated O non-typable (ONT) (Table 2).

### Phenotypic resistance profiles of beta-lactam-resistant GHFF *E. coli*

AST identified resistance to 17 antibiotics from 10 antimicrobial categories across all 13 amoxicillin-resistant GHFF *E. coli* isolates (Table 2). All isolates (*n=*13/13) also exhibited resistance to ampicillin, 69.2 % (*n=*9/13) to tetracycline, 30.8 % (*n=*4/13) to trimethoprim/sulfamethoxazole, 30.8 % (*n=*4/13) to at least one aminoglycoside (streptomycin and/or spectinomycin) and 15.4 % (*n=*2/13) to first- and third-generation cephalosporins (Table 2). One *E. coli* isolate from Sydney (FF993W, ST10 O89:H9) was highly MDR, exhibiting resistance to 10 antimicrobial categories, including carbapenems (imipenem MIC=32 µg ml^−1^), third-generation cephalosporins and fluoroquinolones (Table 2). Overall, 38.5 % (*n=*5/13) of amoxicillin-resistant *E. coli* were MDR, with four of five isolated from wild GHFF and one from a captive GHFF. Resistance to amikacin, gentamycin, colistin and nitrofurantoin was not detected.

### AMR genes and associated elements

Class 1 integrons encoding ARGs were identified in five of 13 *E. coli* isolates (38.5 %), with four of five isolated from wild GHFF and one from a captive GHFF. The most frequent cassette array contained a dihydrofolate reductase gene, *dfrA14* (*n*=3), conferring resistance to trimethoprim, one cassette contained an aminoglycoside adenylyltransferase gene, *aadA1* (conferring spectinomycin and streptomycin resistance), and one cassette contained two genes, *dfrA17* (trimethoprim resistance) and *aadA5* (spectinomycin resistance) (Table S4). In four of five class 1 integrons detected, the typical 3′-conserved segment (*qacE∆1-sul1*) was replaced by an IS*26* transposase and only the integron harbouring *aadA1* carried the full length 3′-conserved segment containing *sul1*. BLASTn searches of the three class 1 integron types found identical sequence matches for each to numerous integrons associated with diverse hosts and *E. coli* strains, including ExPEC strains: GenBank accessions CP038455 and LR130553 (*dfrA14-*IS*26*), MH847038 (*dfrA17-aadA5-*IS*26*) and CP048873 (*aadA1-qacE∆1*).

WGS of *E. coli* isolates identified 16 resistance mechanisms (11 acquired ARGs and five known point mutations in intrinsic ARGs) in addition to ARGs identified in class 1 integrons (ARG profiles for all isolates are provided in Table S4). The highly MDR isolate FF993W (ST10 O89:H9) carried nine acquired ARGs and four known point mutations. The remaining 12 isolates carried between one and five acquired resistance genes or known point mutations. AMR genes and corresponding EUCAST phenotypic antibiotic resistance profiles are shown in Table 2.

Resistance to amoxicillin and ampicillin was predominantly conferred by *bla*_TEM_ genes (92.3 %), *bla*_TEM-1A_ (*n*=1/13) and *bla*_TEM-1B_ (*n*=11/13). A single isolate (ST131) carried a known point mutation in the *ampC* gene promotor (T-32A), conferring resistance to amoxicillin and ampicillin, plus amoxicillin-clavulanic acid, first-generation cephalosporins and intermediate resistance to third-generation cephalosporins. In the highly MDR ST10 O89:H9 isolate, first- and third-generation cephalosporin resistance was associated with *bla*_CTX-M-27_ (100 % identity to GenBank sequence AY156923) and carbapenem resistance was conferred by a New Delhi metallo-beta-lactamase *bla*_NDM-5_ (100 % identity to GenBank sequence JN104597). The *bla*_NDM-5_ gene was carried on an IncX3 plasmid (partial sequence, 45 510 bp, GenBank MT264996) and showed >99.99 % identity to numerous *bla*_NDM-5_ carrying IncX3 plasmids that were predominantly associated with *E. coli* (GenBank MH347484, MG825384 and MG825382), but also *Klebsiella pneumoniae* (GenBank MK628734), *Citrobacter freundii* (GenBank CP024820) and *Proteus mirabilis* (GenBank CP043333). The *E. coli* isolates harbouring highly similar IncX3 plasmids were associated with diverse STs and hosts, including ST48 from geese (GenBank CP034745), ST156 from human blood (GenBank CP048025), ST977 from pork (GenBank MG825382), ST1011 from human faeces (GenBank AP023197) and ST1079 from chicken (GenBank MG825384). The FF993W *bla*_NDM-5_ IncX3 plasmid (GenBank MT264996) did not harbour class 1 integrons or any other resistance genes, but did carry multiple transposases (IS*5*/IS*1182*, IS*30* and ISL*3*).

Eight of nine tetracycline-resistant isolates carried *tet(A*), although the genetic mechanism of tetracycline resistance could not be identified in the ninth isolate FF1155A (ST117). Trimethoprim plus sulfamethoxazole-resistant isolates all carried *sul2* (*n=*4/4), in addition to the *dfrA* genes identified in class 1 integrons. Two of 13 isolates carried *aph(3′′)-Ib* and *aph(6)-Id* conferring streptomycin resistance. In isolate FF993W (ST10 O89:H9), fluoroquinolone resistance was conferred by a combination of four point mutations in *gyrA* (S83L and D87N), *parC* (S80I) and *parE* (L416F), and chloramphenicol resistance by *catA2*. The ST394 isolate carried the *qnrS1* gene but failed to exhibit phenotypic resistance to quinolones or fluoroquinolones.

### VFs and plasmids of GHFF *E. coli* isolates

WGS analysis revealed 21 ExPEC-associated VFs distributed among the 13 GHFF *E. coli,* with the most frequent VFs being *fimH* (100 %, *n=*13/13)*, iss* and *ompT* (69.2 %, *n*=9/13), *sitA* (61.5 %, *n*=8/13), *traT* (53.8 %, *n*=7/13) and *iroN* (46.2 %, *n*=6/13) (Table S4). Less frequently detected VFs included aerobactin (*iutA*/*iucA*), *afa, astA,* colibactin (*clbB*), *cnf1*, *kpsM* II, *papC, sfa*/*foc*, uropathogenic-specific protein (*usp*) and yersiniabactin (*fyuA/irp/ybt*), which were present in less than 40 % of GHFF *E. coli* isolates. Six VFs (*gimB*, *iha, ireA*, *malX, neuC* and *sat*) were not detected. At least one bacteriocin was present in eight isolates (61.5 %), and four of these eight were carrying both colicin and microcin VFs (Table S4). Between one and five plasmids were identified in the GHFF isolates, including IncFIB (69.2 %, *n*=9/13), IncFII (46.2 %, *n*=6/13), IncX (30.8 %, *n*=4/13) and Col (30.8 %, *n*=4/13). The VF and plasmid profiles for all isolates are provided in Table S4.

The carriage of three key ExPEC VFs (*afa, iutA* and *sfa/foc*) by FF1659B (ST73 O22:H1) made it the only GHFF *E. coli* isolate meeting the criteria for ExPEC. A further six isolates (46.2 %) each carried one key ExPEC-associated VF. Based on overall ExPEC VF profiles, the 13 GHFF isolates were assigned to four pathotypes: ExPEC (7.7%, *n=*1/13), ExPEC-potential (46.2 %, *n=*6/13), ExPEC-like (15.4 %, *n=*2/13) and low pathogenicity (30.8 %, *n=*4/13) (Table 2). Of the nine *E. coli* isolates with ExPEC traits, the majority (8/9) were from wild GHFF and one (FF1084) was from a captive GHFF at Mylor (Table 2).

### Phylogenies of *E. coli* with ExPEC traits

### Human-associated ExPEC lineages

GrapeTree phylogenetic analysis placed three of the nine GHFF isolates with ExPEC traits (FF993W, FF1659B and FF1145A) in lineages predominantly comprising human-sourced isolates (Fig. 1a–c). All three GHFF isolates were clustered with very closely related human-sourced ExPEC isolates, with the most closely related isolates having only 13, 11 and 21 cgMLST allelic differences for FF993W, FF1659B and FF1145A respectively. All three GHFF human-associated ExPEC isolates harboured class 1 integrons, and two were considered MDR (FF993W and FF1145A).

The highly MDR *E. coli* isolate FF993W (ST10 O89:H9) was the only O89:H9 serotype isolate in a cluster of predominantly clinical human-sourced O9:H9 serotype isolates (Fig. 1a). The cluster was broadly divided into two sub-clusters harbouring different VF and AMR profiles, with aerobactin, *bla*_CTX-M-15,_ *bla*_OXA-1_ and *dfrA17* present in one sub-cluster, and bacteriocins, *bla*_CTX-M-27,_ *bla*_TEM-1B_ and *dfrA14* generally present in the second sub-cluster containing the GHFF isolate (Fig. 1a). Additionally, three variants of the *bla*_NDM_ gene (*bla*_NDM-1_, *bla*_NDM-5_ and *bla*_NDM-7_) were present in isolates in the second sub-cluster containing FF993W (Fig. 1a). FF993W (ST10 O89:H9) was most closely related to three human isolates, two from Germany (ESC-NA8438AA and ESC_NA8451AA having 13–17 cgMLST allelic differences respectively) and one from the USA (ESC_SA9076AA having 24 cgMLST allelic differences) (Fig. 1a). Both FF993W and ESC_SA9076AA harboured identical IncX3 plasmids carrying *bla*_NDM-5_ and two isolates, one from the Philippines (ESC_GA3189AA) and one from the USA (ESC_SA9085AA), harboured IncX3 plasmids carrying *bla*_NDM-7_ (Fig. 1a). The *bla*_NDM-7_ IncX3 plasmid was almost identical to the *bla*_NDM-5_ IncX3 plasmid carried by FF993W and ESC_SA9076AA, with only two nucleotide differences between the *bla*_NDM_ genes and a 1277 bp IS*30* transposase deletion in the *bla*_NDM-7_ IncX3 plasmid. The five Philippines sourced isolates in the GHFF sub-cluster harboured *bla*_NDM-1_ on a different 27 kbp plasmid, including ESC_GA3189AA, which co-harboured the *bla*_NDM-7_ IncX3 plasmid (Fig. 1a). All cluster isolates carried an IncFIB plasmid and all except one also carried an IncFII plasmid, but carriage of other plasmid types was highly variable (Fig. 1a).

The only isolate to be classified as ExPEC, FF1695B (ST73 O22:H1), was placed in a cluster with six human ExPEC isolates and was most closely related to three Australian-sourced UPEC (11–28 cgMLST allelic differences) (Fig. 1b). All seven cluster isolates harboured highly similar VF profiles (including *afa, clb*, *cnf1, hly, kpsM* II, *sfa/foc, usp* and yersiniabactin), AMR genes (*aadA1, bla*_TEM-1B_ and *sul1*) and plasmids (Col156, IncFIB and IncFII) (Fig. 1b).

The GHFF *E. coli* isolate FF1145A (ST394 O17/O77:H18) belonged to a predominantly human-associated cluster, including one ExPEC, and was most closely related to two human faecal-sourced isolates from Cambodia (21 cgMLST allelic differences) (Fig. 1c). All cluster isolates shared almost identical VF profiles, including *KpSM* II, *lpfA* and *ompT,* whereas AMR and VF profiles were diverse, with the exception of *bla*_TEM-1B_ and *tet(A*) carriage by almost all isolates (Fig. 1c).

#### Human- and animal-associated ExPEC lineages

GrapeTree phylogenetic analysis placed four isolates with ExPEC traits (FF1616, FF1659A, FF1084 and FF1249) in lineages comprised of human- and animal-sourced isolates (Fig. 2a–d). However, all four GHFF isolates belonged to clusters predominantly containing animal-sourced isolates, with at least one human-associated isolate being present in three of four clusters (Fig. 2a–d). The most closely related isolates had 83, 54, 74 and 32 cgMLST allelic differences for FF1616, FF1659A, FF1084 and FF1249 respectively, and are notably less closely related to GHFF isolates in comparison to the three GHFF isolates clustered with human-associated ExPEC (11–21 cgMLST allelic differences).

The FF1616 (ST88 O8:H19) cluster included one human ExPEC and three poultry-sourced isolates showing 83–104 cgMLST allelic differences to FF1616 (Fig. 2a). All ST88 O8:H19 cluster isolates harboured bacteriocins, yersiniabactin and *papC* VFs (Fig. 2a). Four of five cluster isolates harboured IncFIB and IncFII plasmids, but carriage of other plasmid types and ARGs was highly variable (Fig. 2a).

The FF1659A (ST155 ONT:H9) and FF1084 (ST1850 O9:H10) *E. coli* clusters both contained isolates, including ExPEC, from diverse host sources (Fig. 2b, c). The ST155 ONT:H9 cluster isolates showed 54–71 cgMLST allelic differences to FF1659A and all harboured aerobactin, yersiniabactin and bacteriocins (Fig. 2b). ST155 cluster isolates also harboured similar plasmid profiles, but ARG carriage was variable (Fig. 2b). The ST1850 O9:H10 cluster contained two ExPEC isolates, one sourced from human blood and the other from canine urine, showing 86 and 82 cgMLST allelic differences to FF1084 respectively (Fig. 2c). ST1850 cluster isolates showed highly variable VF, ARG and plasmid profiles (Fig. 2c).

FF1249 belonged to a highly virulent ST131 O25:H4 *fimH*22 clade B virotype D cluster, with no isolates harbouring *bla*_CTX_M-15_ or *parC*/*gyrA* allele variants conferring fluoroquinolone resistance (Fig. 2d). Two cluster isolates, FF1249 and the gull isolate (ESC_IA0983AA), carried an *ampC* point mutation (T-32A) conferring resistance to cephalosporins, and no isolates harboured any acquired ARGs (Fig. 2d). The GHFF ST131 cluster contained six canine-sourced ExPEC and one gull faecal isolate, showing 32–46 cgMLST allelic differences to FF1249 (Fig. 2d). The most closely related isolate (32 cgMLST allelic differences) was sourced from canine urine in Australia (ESC_SA4313AA) (Fig. 2d). The ST131 cluster isolates shared almost identical VF profiles, including *ibeA*, *KpsM* II, *usp* and yersiniabactin, and all isolates carried Col156, IncFIB and IncFII plasmids, with one exception (Fig. 2d).

#### Animal-associated ExPEC lineages

GrapeTree phylogenetic analysis placed two isolates with ExPEC traits (FF1155A and FF1140B) in lineages consisting of animal- and environmental-sourced isolates, including ExPEC, but were devoid of human-associated isolates (Fig. 3a and b). These two GHFF isolates were not closely related to cluster isolates, with the most closely related having 108 and 149 cgMLST allelic differences for FF1155A and FF1140B respectively.

Isolate FF1155A (ST117 O85:H18) was clustered with six Ovariable:H18 isolates that showed 108–226 cgMLST allelic differences (Fig. 3a). All ST117 O85:H187 cluster isolates had similar VF profiles, with some carrying bacteriocins and aerobactin, and included one canine UPEC (Fig. 3a). ST117 cluster isolates harboured highly variable ARG and plasmid profiles, with the exception of IncFIB plasmid carriage by six of seven isolates (Fig. 3a).

Isolate FF1140B (ST1673 ONT:H21) belonged to a small cluster containing three isolates from poultry and one from bovine (Fig. 3b). Two of the three poultry isolates were APEC sourced in Australian which showed 169 and 170 cgMLST allelic differences to FF1140B (Fig. 3b). All ST1673 cluster isolates harboured bacteriocins, *hlyF* and *iss*, with aerobactin present in all isolates except the GHFF isolate (FF1140B) (Fig. 3b). ST1673 cluster isolates all harboured IncFIB and IncI1-I plasmids, but carriage of other plasmids and ARGs was variable (Fig. 3b).

#### Phylogenies of *E. coli* with low pathogenicity

Four *E. coli* isolates presented as low pathogenicity, comprising three from wild GHFF (FF1150B, FF1158A and FF1640) and one (FF1091) from a captive GHFF at Mylor. GrapeTree phylogenetic analysis placed all four isolates in lineages containing both human- and animal-sourced isolates,(Fig. 4a–d), with three of four isolates belonging to the ST10 complex (ST10, ST48 and ST7187) (Fig. 4a–c).

The three ST10 complex isolates belonged to clusters predominantly consisting of isolates sourced from animal or human faeces and animal products (Fig. 4a–c). The FF1158A (ST10 O16:H4) isolate was not closely related to other cluster isolates (471–684 cgMLST allelic differences), whereas FF1091 (ST48 O4:H26) and FF1150B (ST7187 ONT:H11) were more closely related to cluster isolates (111–195 and 24–321 cgMLST allelic differences respectively) (Fig. 4a–c). Isolates in the three ST10 complex clusters exhibited highly variable ARG carriage and generally carried few VFs, although all three clusters contained at least one isolate which had acquired considerable VFs (Fig. 4a–c).

The remaining low pathogenic GHFF isolate, FF1640 (ST398 O155:H20), belonged to an ST398 Ovariable:H20 cluster, with isolates showing 95–254 cgMLST allelic differences to FF1640 (Fig. 4d). The cluster contained two ST398 O155:H20 ExPEC isolates, one sourced from human blood and one bovine mastitis (Fig. 4d). Only four ST398 cluster isolates harboured more than one VF, and AMR carriage was highly variable (Fig. 4d).

## Discussion

In this study, antimicrobial-resistant *E. coli* were isolated from 3.5 % of wild and 6.5 % of captive GHFF faecal samples, which is in agreement with studies reporting higher occurrences of AMR determinants (class 1 integrons) in captive GHFF [45] and antimicrobial-resistant *E. coli* in Australian captive wild birds [23]. Of the 13 amoxicillin-resistant *E. coli* isolates from GHFF, two exhibited resistance to at least one human and veterinary CIA (including carbapenems, third-generation cephalosporins and fluoroquinolones) [3, 4]. Bacterial isolates exhibiting resistance to CIAs are classified as priority 1 (critical) antibiotic-resistant bacterial pathogens requiring urgent research and development of new antibiotics [62].

Although this study reported a limited number of beta-lactam-resistant *E. coli* isolates (*n*=13), reflecting the low occurrence (3.8 % overall) in a large sample size of GHFF (*n*=318), the data clearly demonstrate the transmission of antimicrobial-resistant *E. coli* from humans and domestic animals into GHFF.

The presence of antimicrobial-resistant *E. coli* in wild GHFF across all sampled regions were less variable in comparison to the captive GHFF. Overall, the occurrence of resistance to amoxicillin and ampicillin in *E. coli* isolated from wild GHFF was low (3.5 %) and similar to levels of ampicillin resistance previously reported in Australian wild mammals (2.9 %) [63]. As this study selected for amoxicillin-resistant *E. coli* isolates, the occurrence of *E. coli* carrying resistance to non-beta-lactam categories of antimicrobials may be underestimated in GHFF. The low levels of resistance to third-generation cephalosporins and fluoroquinolones, and absence of resistance to colistin in *E. coli* from GHFF correlate with low levels (≤3.0 %) observed in Australian food-producing animals [64, 65], wild mammals [63] and wild birds [23]. However, widespread occurrence of resistance to third-generation cephalosporins and fluoroquinolones has recently been reported in *E. coli* from Australian silver gulls [18].

Of most concern was the detection of a highly MDR *E. coli* isolate from a wild GHFF in Sydney which exhibited resistance to three CIAs, specifically carbapenems (*bla*_NDM-5_), third-generation cephalosporins (*bla*_CTX-M-27_), fluoroquinolones and seven additional categories of antimicrobials. The first *bla*_NDM-5_-producing *E. coli* were reported in 2011 from a human clinical case in the UK, following hospitalization in India [66]. Subsequently, *bla*_NDM-5_ has been reported globally in human clinical *E. coli* isolates, including in Australia [67], and in food-producing animals [68] and companion animals [69, 70]. To date, carbapenemase-producing *E. coli* have not been detected in Australian food-producing [64, 65] or companion animals [71–73] but were detected in Australian silver gulls carrying *E. coli* harbouring *bla*_IMP-4_ and *bla*_OXA-48_ at two sampled locations [18, 20]. This is the first detection of *bla*_NDM-5_ in *E. coli* isolated from non-human hosts in Australia, and to the best of our knowledge, the first report of a carbapenemase-producing *E. coli* in bats globally. Previously, carbapenemase-producing *Klebsiella pneumoniae* (*bla*_OXA-48_ and *bla*_KPC-3_) have been isolated from microbat guano in Algeria [74].

In four of the five class 1 integrons harboured by GHFF *E. coli *, the typical 3′-conserved segment (*qacE∆1-sul1*) was replaced by an IS*26* transposase, which is consistent with reports of an increased prevalence of *IntI1-*ARG*-*IS*26* structured class 1 integrons in *E. coli* from both animals and humans in Australia [48, 75, 76]. The only integron containing the typical 3′-conserved segment (*IntI1-aadA1-qacE∆1-sul1*) was harboured by the ST73 O22:H1 isolate (FF1659B). This integron was also characteristically present in human clinical ST73 ExPEC in an Australian study, including two isolates closely related to the GHFF ST73 isolate [77].

The genetic characterization of antimicrobial-resistant *E. coli* detected in GHFF demonstrates a transmission pathway from humans and/or domestic animals into flying foxes living in urban and/or captive environments. The detection of three isolates belonging to human-associated ExPEC lineages (ST10 O89:H9 and O9:H9, ST73 O22:H1 and ST394 O17/O77:H18) adds to growing evidence for spillover of antimicrobial-resistant pathogenic *E. coli* strains from humans into wildlife [15–18]. ST10 O89:H9 and O9:H9 MDR *E. coli* have also been isolated from wild silver gulls in Australia [18], although the GHFF ST10 O89:H9 isolate was more closely related to MDR human clinical isolates [61], suggesting wild gulls were not the source of the GHFF isolate. ST73 is increasingly associated with human UTIs, and more recently, blood infections [14, 77]. The ST73 ExPEC isolate was detected in a wild GHFF from Lake Macquarie and was very closely related to two human clinical isolates (11 and 21 cgMLST allelic differences) sourced from urine samples at a hospital located approximately 100 km from the GHFF colony [77]. ST394 clonal group A (CGA) is typically a human intestinal pathogen (enteroaggregative *E. coli *), although some lineages are associated with UTIs and bacterial prostatitis [78, 79].

Seven additional GHFF isolates belonged to lineages associated with extraintestinal disease in humans and/or domestic animals (ST88, ST117, ST131, ST155 complex, ST398 and ST1850) [80–83]. These findings indicate the source of these isolates is likely to be domestic animals, but they have the potential to be zoonotic pathogens. Four of the seven STs have been reported as APEC in Australia (ST88, ST117, ST155 and ST1673) [84]. ST117 belongs to the recently described phylogroup G lineage, which is associated with extraintestinal disease primarily in poultry [84], but also in domestic animals and humans [85]. ST131 O25:H4 is a globally distributed ExPEC clonal lineage consisting of three distinct clades (A, B and C) [2, 58]. The GHFF ST131 clade B isolate was detected in a wild GHFF from Sydney, New South Wales, and was clustered with six canine-sourced ExPEC isolates. Interestingly, the most closely related isolate (32 cgMLST allelic differences) was a canine UPEC, also sourced from New South Wales, Australia. Clinical infections with ST131 from diverse clades, including human-associated lineages, have been reported in companion dogs in Australia [72]. These findings indicate a possible transmission pathway from a companion dog to wild GHFF and suggest dogs may facilitate spillover of ST131 ExPEC into Australian wildlife.

While transmission pathways were not part of this study, the most likely acquisition source of antimicrobial-resistant *E. coli* by wild GHFF is exposure to water contaminated by effluent and runoff [86]. Flying foxes are found either in flight or in elevated vegetation and exhibit an unusual ‘dipping’ behaviour to obtain drinking water. GHFF ‘dip’ or skim across the surface of a large freshwater body (lake, pond, dam or river) whilst in flight and return to a roost to lick their wet fur to intake water, and any microbes it carries. Further studies encompassing sampling of urban waters in the vicinity of GHFF colonies would be required to identify transmission pathways in wild GHFF. Several studies have documented the acquisition of MDR *E. coli* by captive animals, with potential sources including human wildlife carers, domestic animals and other wildlife in captivity [22, 23, 87].

The majority of studies examining AMR and pathogenic *E. coli* in wildlife largely focus on the role of wildlife as reservoirs of AMR and zoonotic bacteria [86, 88]. Equally relevant is the potential for anthropogenically derived bacteria to negatively impact or cause disease in wildlife (reverse zoonosis or zooanthroponosis) [89]. It is currently unknown if the acquisition of pathogenic *E. coli* strains from human and domestic animal sources by GHFF can cause disease in flying foxes. *E. coli* isolates with ExPEC virulence characteristics have been isolated from the internal organs of fruit bats in the Republic of Congo, although it was unknown if carriage was associated with clinical disease [33].

Every year thousands of sick and injured flying foxes enter wildlife hospitals and wildlife rehabilitation facilities in Australia [90] with many requiring antimicrobial therapy. Amoxicillin, amoxicillin-clavulanic acid and enrofloxacin (a veterinary fluoroquinolone) are all commonly prescribed to flying foxes in care [91], and resistance to all three was detected in GHFF in this study. Antimicrobial administration to GHFF may select for antimicrobial-resistant *E. coli* and result in a poor response to treatment, increase the risk of developing secondary ExPEC infections and reduce the prognosis for recovery. Selecting for resistant isolates also increases the potential for their dissemination to other flying foxes in care, their human carers and into the environment after release from captivity. The emergence of anthropogenic AMR and *E. coli* exhibiting ExPEC characteristics in GHFF is yet another threat to this already threatened flying fox species, potentially exacerbated by heat stress events, habitat loss and food shortages, which are resulting in increasing numbers of GHFF entering care each year [90, 92].

The zoonotic and public health risks associated with antimicrobial-resistant *E. coli* carriage by wild GHFF is low (3.5 % occurrence), although it must be considered that urban GHFF colonies typically contain 10 000 to 20 000 individuals, which equates to approximately 350–700 GHFF per colony that may disseminate potentially zoonotic *E. coli* in urban environments [93]. The detection of one MDR carbapenemase-producing human clinical isolate (ST10 O89:H9), one confirmed human ExPEC isolate (ST73) and seven additional isolates with ExPEC potential indicates GHFF can harbour antimicrobial-resistant *E. coli* capable of causing extraintestinal infections in humans.

## Conclusion

This study has demonstrated the transmission of anthropogenic *E. coli* harbouring diverse AMR mechanisms and ExPEC virulence traits to GHFF in urban and captive environments. This is the first detection of *bla*_NDM-5_ carrying carbapenem-resistant *E. coli* from a non-human host in Australia, suggesting wild GHFF may act as vectors of carbapenem resistance. This study also suggests GHFF may be potential reservoirs for antimicrobial-resistant human-associated ExPEC lineages, providing opportunities for zoonosis to occur. The highly mobile nature of GHFF increases the potential to disseminate *E. coli* over wide areas, including urban environments. This study highlights the importance of a One Health approach, incorporating human, animal and ecosystem health, to investigating the prevalence of AMR and zoonotic diseases. Adopting surveillance methods which incorporate genetic characterization of antimicrobial-resistant isolates can identify potential zoonotic and zooanthroponotic risks, benefitting both public health and flying fox health.

## Supplementary Data

Supplementary material 1

## References

[1] Manges AR, Johnson JR, Foxman B, O'Bryan TT, Fullerton KE (2001). Widespread distribution of urinary tract infections caused by a multidrug-resistant *Escherichia coli* clonal group. New Engl J Med.

[2] Petty NK, Zakour NLB, Stanton-Cook M, Skippington E, Totsika M (2014). Global dissemination of a multidrug resistant *Escherichia coli* clone. Proc Natl Acad Sci.

[3] WHO (2019). Critically important antimicrobials for human medicine, 6th revision 2018. World health organisation (who). https://apps.who.int/iris/bitstream/handle/10665/312266/9789241515528-eng.pdf?ua=1..

[4] OIE (2018). OIE list of antimicrobial agents of veterinary importance. the world organisation for animal health (OIE). https://www.oie.int/fileadmin/Home/eng/Our_scientific_expertise/docs/pdf/AMR/A_OIE_List_antimicrobials_July2019.pdf.

[5] Partridge SR, Kwong SM, Firth N, Jensen SO (2018). Mobile genetic elements associated with antimicrobial resistance. Clin Microbiol Rev.

[6] Stokes HW, Gillings MR (2011). Gene flow, mobile genetic elements and the recruitment of antibiotic resistance genes into gram-negative pathogens. FEMS Microbiol Rev.

[7] Hall RM, Collis CM (1995). Mobile gene cassettes and integrons: capture and spread of genes by site‐specific recombination. Mol Microbiol.

[8] Kaper JB, Nataro JP, Mobley HL (2004). Pathogenic *Escherichia coli*. Nat Rev Microbiol.

[9] Köhler C-D, Dobrindt U (2011). What defines extraintestinal pathogenic *Escherichia coli*?. Int J Med Microbiol.

[10] Wirth T, Falush D, Lan R, Colles F, Mensa P (2006). Sex and virulence in *Escherichia coli*: an evolutionary perspective. Mol Microbiol.

[11] Riley L (2014). Pandemic lineages of extraintestinal pathogenic *Escherichia coli*. Clin Microbiol Infect.

[12] Skjøt-Rasmussen L, Olsen S, Jakobsen L, Ejrnaes K, Scheutz F (2013). *Escherichia coli* clonal group A causing bacteraemia of urinary tract origin. Clin Microbiol Infect.

[13] Nicolas-Chanoine M-H, Bertrand X, Madec J-Y (2014). *Escherichia coli* ST131, an intriguing clonal group. Clin Microbiol Rev.

[14] Alhashash F, Wang X, Paszkiewicz K, Diggle M, Zong Z (2016). Increase in bacteraemia cases in the East Midlands region of the UK due to MDR *Escherichia coli* ST73: high levels of genomic and plasmid diversity in causative isolates. J Antimicrob Chemother.

[15] P-L H, Lo W-U LEL, Law PY, Leung SM, Wang Y (2015). Clonal diversity of CTX-M-producing, multidrug-resistant *Escherichia coli* from rodents. J Med Microbiol.

[16] Hasan B, Olsen B, Alam A, Akter L, Å M (2015). Dissemination of the multidrug-resistant extended-spectrum β-lactamase-producing *Escherichia coli* O25b-ST131 clone and the role of house crow (*Corvus splendens*) foraging on hospital waste in Bangladesh. Clin Microbiol Infect.

[17] Mora A, García-Peña FJ, Alonso MP, Pedraza-Diaz S, Ortega-Mora LM (2018). Impact of human-associated *Escherichia coli* clonal groups in Antarctic pinnipeds: presence of ST73, ST95, ST141 and ST131. Sci Rep.

[18] Mukerji S, Stegger M, Truswell AV, Laird T, Jordan D (2019). Resistance to critically important antimicrobials in Australian silver gulls (*Chroicocephalus novaehollandiae*) and evidence of anthropogenic origins. J Antimicrob Chemother.

[19] Guenther S, Ewers C, Wieler LH (2011). Extended-spectrum beta-lactamases producing *E. coli* in wildlife yet another form of environmental pollution?. Front Microbiol.

[20] Dolejska M, Masarikova M, Dobiasova H, Jamborova I, Karpiskova R (2016). High prevalence of *Salmonella* and IMP-4-producing Enterobacteriaceae in the silver gull on Five Islands, Australia. J Antimicrob Chemother.

[21] Skurnik D, Ruimy R, Andremont A, Amorin C, Rouquet P (2006). Effect of human vicinity on antimicrobial resistance and integrons in animal faecal *Escherichia coli*. J Antimicrob Chemother.

[22] Kinjo T, Minamoto N, Sugiyama M, Sugiyama Y (1992). Comparison of antimicrobial resistant *Escherichia coli* in wild and captive Japanese serows. J Vet Med Sci.

[23] Blyton MD, Pi H, Vangchhia B, Abraham S, Trott DJ (2015). Genetic structure and antimicrobial resistance of *Escherichia coli* and cryptic clades in birds with diverse human associations. Appl Environ Microbiol.

[24] SS M, Urdahl AM, Madslien K, Sunde M, Nesse LL (2018). What does the Fox say? monitoring antimicrobial resistance in the environment using wild red foxes as an indicator. PLoS One..

[25] Wang J, Ma Z-B ZZ-L, Yang X-W, Huang Y, Liu J-H (2017). The role of wildlife (wild birds) in the global transmission of antimicrobial resistance genes. Zool Res.

[26] Cooper LN, Cretekos CJ, Sears KE (2012). The evolution and development of mammalian flight. Wiley Interdiscip Rev Dev Biol.

[27] Tidemann CR, Nelson JE (2004). Long-distance movements of the grey-headed flying fox (*Pteropus poliocephalus*). J Zool.

[28] Welbergen JA, Meade J, Field HE, Edson D, McMichael L (2020). Extreme mobility of the world’s largest flying mammals creates key challenges for management and conservation. BMC Biol.

[29] Burgin CJ, Colella JP, Kahn PL, Upham NS (2018). How many species of mammals are there?. J Mammal.

[30] Teeling EC, Springer MS, Madsen O, Bates P, O'brien SJ (2005). A molecular phylogeny for bats illuminates biogeography and the fossil record. Science.

[31] Iovine RD, Dejuste C, Miranda F, Filoni C, Bueno MG (2015). Isolation of *Escherichia coli* and *Salmonella* spp. from free-ranging wild animals. Braz J Microbiol.

[32] Pinus M, Müller H (1980). Enterobacteria of bats (Chiroptera). Zentralbl Bakteriol A.

[33] Nowak K, Fahr J, Weber N, Lübke-Becker A, Semmler T (2017). Highly diverse and antimicrobial susceptible *Escherichia coli* display a naïve bacterial population in fruit bats from the Republic of Congo. PLoS One.

[34] Klite P (1965). Intestinal bacterial flora and transit time of three Neotropical bat species. J Bacteriol.

[35] Moreno G, Lopes C, Seabra E, Pavan C, Correa A (1975). Bacteriological study of the intestinal flora of bats (*Desmodus rotundus*). Arq Inst Biol.

[36] Heard DJ, De Young JL, Goodyear B, Ellis GA (1997). Comparative rectal bacterial flora of four species of flying fox (*Pteropus* sp. J Zoo Wildl Med.

[37] Benavides J, Shiva C, Virhuez M, Tello C, Appelgren A (2018). Extended‐spectrum beta‐lactamase‐producing *Escherichia coli* in common vampire bats *Desmodus rotundus* and livestock in Peru. Zoonoses Publ Health.

[38] Nowakiewicz A, Zięba P, Gnat S, Trościańczyk A, Osińska M (2020). Bats as a reservoir of resistant *Escherichia coli*: A methodical view. Can we fully estimate the scale of resistance in the reservoirs of free-living animals? Res Vet Sci.

[39] Garcês A, Correia S, Amorim F, Pereira JE, Igrejas G (2019). First report on extended-spectrum beta-lactamase (ESBL) producing *Escherichia coli* from European free-tailed bats (*Tadarida teniotis*) in Portugal: A one-health approach of a hidden contamination problem. J Hazard Mater.

[40] Nguema M, Philippe P, Onanga R, Atome N, Roger G (2020). Characterization of ESBL-producing enterobacteria from fruit bats in an unprotected area of Makokou, Gabon. Microorg.

[41] Oluduro AO (2012). Antibiotic-resistant commensal *Escherichia coli* in faecal droplets from bats and poultry in Nigeria. Vet Ital.

[42] Graves S, Kennelly-Merrit S, Tidemann C, Rawlinson P, Harvey K (1988). Antibiotic-Resistance patterns of enteric bacteria of wild mammals on the Krakatau islands and West Java, Indonesia. Philos Trans R Soc Lond B Biol Sci.

[43] Parry‐Jones K, Augee M (2001). Factors affecting the occupation of a colony site in Sydney, New South Wales by the Grey‐headed Flying‐fox *Pteropus poliocephalus* (Pteropodidae). Austral Ecol.

[44] Currey K, Kendal D, Van der Ree R, Lentini PE (2018). Land manager perspectives on conflict mitigation strategies for urban flying-fox camps. Divers.

[45] McDougall F, Boardman W, Gillings M, Power M (2019). Bats as reservoirs of antibiotic resistance determinants: A survey of class 1 integrons in Grey-headed Flying Foxes (*Pteropus poliocephalus*). Infect Genet Evol.

[46] Matuschek E, Brown DF, Kahlmeter G (2014). Development of the EUCAST disk diffusion antimicrobial susceptibility testing method and its implementation in routine microbiology laboratories. Clin Microbiol Infect.

[47] Magiorakos AP, Srinivasan A, Carey R, Carmeli Y, Falagas M (2012). Multidrug‐resistant, extensively drug‐resistant and pandrug‐resistant bacteria: an international expert proposal for interim standard definitions for acquired resistance. Clin Microbiol Infect.

[48] Dawes FE, Kuzevski A, Bettelheim KA, Hornitzky MA, Djordjevic SP (2010). Distribution of class 1 integrons with IS*26-*mediated deletions in their 3′-conserved segments in *Escherichia coli* of human and animal origin. PLoS One.

[49] Bankevich A, Nurk S, Antipov D, Gurevich AA, Dvorkin M (2012). SPAdes: a new genome assembly algorithm and its applications to single-cell sequencing. J Comput Biol.

[50] Zankari E, Hasman H, Cosentino S, Vestergaard M, Rasmussen S (2012). Identification of acquired antimicrobial resistance genes. J Antimicrob Chemother.

[51] Beghain J, Bridier-Nahmias A, Le Nagard H, Denamur E, Clermont O (2018). ClermonTyping: an easy-to-use and accurate in silico method for *Escherichia* genus strain phylotyping. Microb Genomics.

[52] Roer L, Tchesnokova V, Allesøe R, Muradova M, Chattopadhyay S (2017). Development of a web tool for *Escherichia coli* subtyping based on *fimH* alleles. J Clin Microbiol.

[53] Zhou Z, Alikhan N-F, Mohamed K, Fan Y, Achtman M (2020). The EnteroBase user’s guide, with case studies on *Salmonella* transmissions, *Yersinia pestis* phylogeny, and *Escherichia* core genomic diversity. Genome Res.

[54] Joensen KG, Scheutz F, Lund O, Hasman H, Kaas RS (2014). Real-time whole-genome sequencing for routine typing, surveillance, and outbreak detection of verotoxigenic *Escherichia coli*. J Clin Microbiol.

[55] Chen L, Zheng D, Liu B, Yang J, VFDB JQ (2016). 2016: hierarchical and refined dataset for big data analysis—10 years on. Nucleic Acids Res.

[56] Johnson JR, Kuskowski MA, Owens K, Gajewski A, Winokur PL (2003). Phylogenetic origin and virulence genotype in relation to resistance to fluoroquinolones and/or extended-spectrum cephalosporins and cephamycins among *Escherichia coli* isolates from animals and humans. J Infect Dis.

[57] Pitout J (2012). Extraintestinal pathogenic *Escherichia coli*: a combination of virulence with antibiotic resistance. Front Microbiol.

[58] Zakour NLB, Alsheikh-Hussain AS, Ashcroft MM, NTK N, Roberts LW (2016). Sequential acquisition of virulence and fluoroquinolone resistance has shaped the evolution of *Escherichia coli* ST131. MBio.

[59] Carattoli A, Zankari E, García-Fernández A, Larsen MV, Lund O (2014). *In silico* detection and typing of plasmids using PlasmidFinder and plasmid multilocus sequence typing. Antimicrob Agents Chemother.

[60] Zhou Z, Alikhan N-F, Sergeant MJ, Luhmann N, Vaz C (2018). GrapeTree: visualization of core genomic relationships among 100,000 bacterial pathogens. Genome Res.

[61] Argimón S, Masim MA, Gayeta JM, Lagrada ML, Macaranas PK (2020). Integrating whole-genome sequencing within the National antimicrobial resistance surveillance program in the Philippines. Nature Comm.

[62] WHO (2017). Global priority list of antibiotic-resistant bacteria to guide research, discovery, and developmentof new antibiotics. World health organisation (who). https://www.who.int/medicines/publications/WHO-PPL-Short_Summary_25Feb-ET_NM_WHO.pdf?ua=1.

[63] Sherley M, Gordon DM, Collignon PJ (2000). Variations in antibiotic resistance profile in Enterobacteriaceae isolated from wild Australian mammals. Environ Microbiol.

[64] Abraham S, Jordan D, Wong HS, Johnson JR, Toleman MA (2015). First detection of extended-spectrum cephalosporin-and fluoroquinolone-resistant *Escherichia coli* in Australian food-producing animals. J Glob Antimicrob Resist.

[65] Kidsley AK, Abraham S, Bell JM, O'Dea M, Laird TJ (2018). Antimicrobial susceptibility of *Escherichia coli* and *Salmonella* spp. isolates from healthy pigs in Australia: results of a pilot national survey. Front Microbiol.

[66] Hornsey M, Phee L, Wareham DW (2011). A novel variant, NDM-5, of the New Delhi metallo-β-lactamase in a multidrug-resistant *Escherichia coli* ST648 isolate recovered from a patient in the United Kingdom. Antimicrob Agents Chemother.

[67] Wailan AM, Paterson DL, Kennedy K, Ingram PR, Bursle E (2016). Genomic characteristics of NDM-producing Enterobacteriaceae isolates in Australia and their blaNDM genetic contexts. Antimicrob Agents Chemother.

[68] Yaici L, Haenni M, Saras E, Boudehouche W, Touati A (2016). blaNDM-5-carrying IncX3 plasmid in *Escherichia coli* ST1284 isolated from raw milk collected in a dairy farm in Algeria. J Antimicrob Chemother.

[69] Yousfi M, Mairi A, Bakour S, Touati A, Hassissen L (2015). First report of NDM-5-producing *Escherichia coli* ST1284 isolated from dog in Bejaia, Algeria. New Microbes New Infect.

[70] Hong JS, Song W, Park H-M, J-Y O, Chae J-C (2019). First detection of New Delhi metallo-β-Lactamase-5-producing *Escherichia coli* from companion animals in Korea. Microb Drug Resist.

[71] Saputra S, Jordan D, Mitchell T, San Wong H, Abraham RJ (2017). Antimicrobial resistance in clinical *Escherichia coli* isolated from companion animals in Australia. Vet Microbiol.

[72] Kidsley AK, White RT, Beatson SA, Saputra S, Schembri MA (2020). Companion animals are spillover hosts of the multidrug-resistant human extraintestinal *Escherichia coli* pandemic clones ST131 and ST1193. Front Microbiol.

[73] Rusdi B, Laird T, Abraham R, Ash A, Robertson ID (2018). Carriage of critically important antimicrobial resistant bacteria and zoonotic parasites amongst cAMP dogs in remote Western Australian Indigenous communities. Sci Rep.

[74] Gharout-Sait A, Touati A, Ahmim M, Brasme L, Guillard T (2019). Occurrence of carbapenemase-producing *Klebsiella pneumoniae* in bat guano. Microb Drug Resist.

[75] Hastak P, Cummins ML, Gottlieb T, Cheong E, Merlino J (2020). Genomic profiling of *Escherichia coli* isolates from bacteraemia patients: a 3-year cohort study of isolates collected at a Sydney teaching hospital. Microb Genomics.

[76] Reid CJ, Wyrsch ER, Chowdhury PR, Zingali T, Liu M (2017). Porcine commensal *Escherichia coli*: a reservoir for class 1 integrons associated with IS*26*. Microb Genomics.

[77] Bogema D, McKinnon J, Liu M, Hitchick N, Miller N (2020). Whole-genome analysis of extraintestinal *Escherichia coli* sequence type 73 from a single hospital over a 2 year period identified different circulating clonal groups. Microb Genomics.

[78] Wallace-Gadsden F, Johnson JR, Wain J, Okeke IN (2007). Enteroaggregative *Escherichia coli* related to uropathogenic clonal group A. Emerg Infect Dis.

[79] Krieger JN, Thumbikat P, Mulvey MA, Klumpp D, Stapleton AE (2017). 7 Bacterial prostatitis: bacterial virulence, clinical outcomes, and new directions. Urinary Tract Infections: Molecular Pathogenesis and Clinical Management.

[80] Manges AR, Geum HM, Guo A, Edens TJ, Fibke CD (2019). Global extraintestinal pathogenic *Escherichia coli* (ExPEC) lineages. Clin Microbiol Rev.

[81] Maluta RP, Logue CM, Casas MRT, Meng T, Guastalli EAL (2014). Overlapped sequence types (STs) and serogroups of avian pathogenic (APEC) and human extra-intestinal pathogenic (ExPEC) *Escherichia coli* isolated in Brazil. PLoS One.

[82] Manges A (2016). *Escherichia coli* and urinary tract infections: the role of poultry-meat. Clin Microbiol Infect.

[83] Manges AR, Harel J, Masson L, Edens TJ, Portt A (2015). Multilocus sequence typing and virulence gene profiles associated with *Escherichia coli* from human and animal sources. Foodborne Pathog Dis.

[84] Cummins ML, Reid CJ, Chowdhury PR, Bushell RN, Esbert N (2019). Whole genome sequence analysis of Australian avian pathogenic *Escherichia coli* that carry the class 1 integrase gene. Microb Genomics.

[85] Clermont O, Dixit OV, Vangchhia B, Condamine B, Dion S (2019). Characterization and rapid identification of phylogroup G in *Escherichia coli*, a lineage with high virulence and antibiotic resistance potential. Environ Microbiol.

[86] Arnold KE, Williams NJ, Bennett M (2016). ‘Disperse abroad in the land’: the role of wildlife in the dissemination of antimicrobial resistance. Biol Lett.

[87] Ahmed AM, Motoi Y, Sato M, Maruyama A, Watanabe H (2007). Zoo animals as reservoirs of gram-negative bacteria harboring integrons and antimicrobial resistance genes. Appl Environ Microbiol.

[88] Vittecoq M, Godreuil S, Prugnolle F, Durand P, Brazier L (2016). Antimicrobial resistance in wildlife. J Appl Ecol.

[89] Messenger AM, Barnes AN, Gray GC (2014). Reverse zoonotic disease transmission (zooanthroponosis): a systematic review of seldom-documented human biological threats to animals. PLoS One.

[90] Mo M, Roache M, Haering R, Kwok A (2020). Using wildlife carer records to identify patterns in flying-fox rescues: a case study in New South Wales, Australia. Pac Conserv Biol..

[91] Bodley K, Vogelnest L, Portas T (2019). Appendix 4. Drug formulary. Current Therapy in Medicine of Australian Mammals.

[92] Scheelings TF, Frith SE (2015). Anthropogenic Factors Are the Major Cause of Hospital Admission of a Threatened Species, the Grey-Headed Flying Fox (*Pteropus poliocephalus*), in Victoria, Australia. PLoS One.

[93] Páez DJ, Restif O, Eby P, Plowright RK (1745). Optimal foraging in seasonal environments: implications for residency of Australian flying foxes in food-subsidized urban landscapes. Phil Trans R Soc B Biol Sci.

