# Supplementary material for: Characterization of beta-lactam-resistant Escherichia coli from Australian fruit bats indicates anthropogenic origins
Source: Microb Genom. 2021 May 5;7(5):000571. doi: 10.1099/mgen.0.000571 (PMC8209733; doi:10.1099/mgen.0.000571)
Supplement: Supplementary material 1 [file mgen-7-0571-s001.pdf]

Characterisation of beta-lactam resistant *Escherichia coli* from Australian fruit bats indicates anthropogenic origins

Fiona K. McDougall, Wayne S.J. Boardman and Michelle L. Power

Supplementary tables

Table S1. Antibiotic discs used for EUCAST susceptibility testing.

Antibiotic and disc content (µg)	OXOID disc	Antibiotic category and generation	Breakpoint
Ampicillin (10 µg)	AMP10	ß-lactam/Penicillins	EUCAST
Amoxicillin/Clavulanic Acid (30 µg)	AMC30	ß-lactam/Penicillins + ß-lactamase inhibitors	EUCAST
Cephalexin (30 µg)	CL30	ß-lactam/Cephalosporins/1st Generation (Non-extended spectrum cephalosporin)	EUCAST
Cefazolin (30 µg)	KZ30	ß-lactam/Cephalosporins/1st Generation (Non-extended spectrum cephalosporin)	CLSI
Cefotaxime (5 µg)	CTX5	ß-lactam/Cephalosporins/3rd Generation (Extended-spectrum cephalosporin)	EUCAST
Imipenem (10 µg)	IPM10	ß-lactam/Carbapenems	EUCAST
Meropenem (10 µg)	MEM10	ß-lactam/Carbapenems	EUCAST
Nalidixic acid (30 µg)	NA30	Quinolones-Fluoroquinolones/ 1st Generation Quinolone	CLSI
Ciprofloxacin (5 µg)	CIP5	Quinolones-Fluoroquinolones/ 2nd Generation Fluoroquinolone	EUCAST
Trimethoprim (5 µg)	W5	Trimethoprim (Folate pathway inhibitors)	EUCAST
Trimethoprim/Sulfamethoxazole (30 µg)	SXT30	Trimethoprim + Sulfonamide (Folate pathway inhibitors)	EUCAST
Amikacin (30 µg)	AK30	Aminoglycosides	EUCAST
Gentamycin (10 µg)	CN10	Aminoglycosides	EUCAST
Streptomycin (25 µg)	S25	Aminoglycosides	NA*
Spectinomycin (25 µg)	SH25	Aminoglycosides	NA*
Chloramphenicol (30 µg)	C30	Phenicols	EUCAST
Nitrofurantoin (200 µg)	F200	Nitrofurantoin	NA*
Tetracycline (30 µg)	TE30	Tetracyclines	CLSI

* NA = Not Available: Breakpoint criteria unavailable from EUCAST or CLSI tables.

Table S2. Individual BioSample accession numbers under NCBI Sequence Read Archive (SRA) BioProject ID PRJNA606529, EnteroBase Barcodes and GenBank accession numbers for class 1 integrons and IncX3 plasmid from 13 antimicrobial resistant *E. coli* isolated from GHFF.

Isolate Name	BioSample accession No.	EnteroBase Barcode	Integron GenBank accession No.	IncX3 GenBank accession No.
FF993W	SAMN14096471	ESC_QA8772AA	MT241250	MT264996
FF1084	SAMN14096482	ESC_QA8770AA	-	-
FF1091	SAMN14096483	ESC_QA8771AA	MT241251	-
FF1140B	SAMN14096472	ESC_QA8776AA	-	-
FF1145A	SAMN14096473	ESC_QA8773AA	MT241252	-
FF1150B	SAMN14096479	ESC_QA8780AA	-	-
FF1155A	SAMN14096480	ESC_QA8774AA	-	-
FF1158A	SAMN14096481	ESC_QA8775AA	-	-
FF1249	SAMN14096474	ESC_QA8777AA	-	-
FF1616	SAMN14096475	ESC_QA8779AA	-	-
FF1640	SAMN14096476	ESC_QA8778AA	-	-
FF1659A	SAMN14096477	ESC_PA8159AA	MT241253	-
FF1659B	SAMN14096478	ESC_PA7389AA	MT241254	-

Table S3. URL links to interactive versions of GrapeTree cgMLST phylogeny files for GHFF *E. coli* and related isolates in EnteroBase.

Isolate ST and serotype	Isolate ID	URL link
ST10 O16:H48	FF1158A	https://achtman-lab.github.io/GrapeTree/MSTree_holder.html?tree=https://github.com/Fiona-McDougall/GHFF-Phylogenetics/blob/master/ST10_O16_H48_GrapeTree_ms_tree.json
ST10 O89:H9	FF993W	https://achtman-lab.github.io/GrapeTree/MSTree_holder.html?tree=https://github.com/Fiona-McDougall/GHFF-Phylogenetics/blob/master/ST10_O89_H9_GrapeTree_ms_tree.json
ST48 O4:H26	FF1091	https://achtman-lab.github.io/GrapeTree/MSTree_holder.html?tree=https://github.com/Fiona-McDougall/GHFF-Phylogenetics/blob/master/ST48_O4_H26_GrapeTree_ms_tree.json
ST73 O22:H1	FF1659B	https://achtman-lab.github.io/GrapeTree/MSTree_holder.html?tree=https://github.com/Fiona-McDougall/GHFF-Phylogenetics/blob/master/ST73_O22_H1_GrapeTree_ms_tree.json
ST88 O8:H19	FF1616	https://achtman-lab.github.io/GrapeTree/MSTree_holder.html?tree=https://github.com/Fiona-McDougall/GHFF-Phylogenetics/blob/master/ST88_O8_H19_GrapeTree_ms_tree.json
ST117 O85:H18	FF1155A	https://achtman-lab.github.io/GrapeTree/MSTree_holder.html?tree=https://github.com/Fiona-McDougall/GHFF-Phylogenetics/blob/master/ST117_O85_H18_GrapeTree_ms_tree.json
ST131 O25:H4	FF1249	https://achtman-lab.github.io/GrapeTree/MSTree_holder.html?tree=https://github.com/Fiona-McDougall/GHFF-Phylogenetics/blob/master/ST131_O25_H4_GrapeTree_ms_tree.json

ST155 ONT:H9	FF1659A	https://achtman-lab.github.io/GrapeTree/MSTree_holder.html?tree=https://github.com/Fiona-McDougall/GHFF-Phylogenetics/blob/master/ST155_ONT_H9_GrapeTree_ms_tree.json
ST394 O17/O77:H18	FF1145A	https://achtman-lab.github.io/GrapeTree/MSTree_holder.html?tree=https://github.com/Fiona-McDougall/GHFF-Phylogenetics/blob/master/ST394_O17_O77_H18_GrapeTree_ms_tree.json
ST398 O155:H20	FF1640	https://achtman-lab.github.io/GrapeTree/MSTree_holder.html?tree=https://github.com/Fiona-McDougall/GHFF-Phylogenetics/blob/master/ST398_O155_H20_GrapeTree_ms_tree.json
ST1673 ONT:H21	FF1140B	https://achtman-lab.github.io/GrapeTree/MSTree_holder.html?tree=https://github.com/Fiona-McDougall/GHFF-Phylogenetics/blob/master/ST1673_ONT_H21_GrapeTree_ms_tree.json
ST1850 O9:H10	FF1084	https://achtman-lab.github.io/GrapeTree/MSTree_holder.html?tree=https://github.com/Fiona-McDougall/GHFF-Phylogenetics/blob/master/ST1850_09_H10_GrapeTree2_ms_tree.json
ST7187 ONT:H11	FF1150B	https://achtman-lab.github.io/GrapeTree/MSTree_holder.html?tree=https://github.com/Fiona-McDougall/GHFF-Phylogenetics/blob/master/ST7187_ONT_H11_GrapeTree_ms_tree.json

Table S4. Antimicrobial resistance determinants, virulence factor and plasmid profiles for GHFF *E. coli* isolates. ARG, Antimicrobial resistance genes. NT, Not typable. VF, Virulence factor.

Isolate ID	Class 1 integron associated ARGs	Acquired ARGs	Point mutations in intrinsic ARGs	Key ExPEC VFs	ExPEC VFs	Additional VFs	Plasmids
FF993W	*dfrA14*-IS*26*	*aph(3")-Ib, aph(6)-Id, bla*TEM-1B*, bla*CTX-M-27*, bla*NDM-*5, catA2, sul2, tet(A)*	*parC* (S80I) *parE* (L416F) *gyrA* (S83L) *gyrA* (D87N)	-	*fimH, sitA, traT*	*cma, cvaC*	IncFIB, IncFII, IncX3
FF1084	-	*bla*TEM-1B*, tet(A)*	-	*iucA/iutA*	*fimH, hlyF, iroN, iss, ompT, sitA, traT, usp*	*cia, cvaC, mchBCF/mcmA*	IncFIA, IncFIB, IncFIC(FII)
FF1091	*dfrA17-aadA5*-IS*26*	*bla*TEM-1B*, tet(A), sul2*	-	-	*fimH*	-	Col156, IncHI2, IncHI2A, p0111
FF1140B	-	*bla*TEM-1B	-	-	*fimH, hlyF iroN, iss, ompT, sitA*	*cia, cma, cvaC, lpfA*	IncFIB, IncFII, IncI1
FF1145A	*dfrA14*-IS*26*	*bla*TEM-1B*, sul2, qnrS1*, tet(A)*	-	*kpsM* II	*fimH, astA, ompT*	*air/eilA, chuA, gtrAB, lpfA*	IncY
FF1150B	-	*bla*TEM-1B*, tet(A)*	-	-	*fimH, astA, iss*	-	IncN, p0111
FF1155A	-	*bla*TEM-1A	-	*iucA/iutA*	*fimH, hlyF, iroN, iss, ompT, pic, sitA, traT, vat*	*cea, chuA, cma, cvaC, lpfA, microcin B17*, *mchF, sinH*	IncFIB, IncFIC(FII), IncFII, IncX1, IncX4
FF1158A	-	*bla*TEM-1B*, tet(A)*	-	-	*fimH, iss, ompT*	*gtrAB*	IncFIB(K), IncHI1B, p0111

FF1249	-	-	*ampC* (T-32A)	*kpsM* II	*fimH, fyuA/irp/ybt, ibeA, iss, ompT, sitA traT, usp*	*chuA, senB*	Col156, IncFIB, IncFIB(H89), IncFII
FF1616	-	*bla*TEM-1B*, tet(A)*	-	*papC*	*fimH, fyuA/irp/ybt, hlyF, iroN, iss, ompT, sitA, traT*	*cia, cma, lpfA*	IncFIB, IncFII
FF1640	-	*aph(3')-Ia*, bla*TEM-1B*, tet(A)*	-	-	*fimH, astA*	-	ColRNAI, IncX1
FF1659A	*dfrA14*-IS*26*	*aph(3'')-Ib, aph(6)-Id, bla*TEM-1B*, sul2*	-	*iucA/iutA*	*fimH, fyuA/irp/ybt, hlyF, iroN, iss, ompT, sitA, traT, tsh*	*cia, cvaC mchF, lpfA*	IncFIA, IncFIB, IncFIC(FII), IncI1
FF1659B	*aadA1-qacE∆1-sul1*	*bla*TEM-1B	-	*afaD kpsM* II *sfa/foc*	*fimH, clbB, cnf1, fyuA/irp/ybt, hlyABCD, iroN, iss, ompT, pic, sitA, traT, usp, vat*	*cea, chuA, microcin H47, senB*	Col156, Col440II, IncFIB, IncFII, IncX4

* Resistance gene identified in whole genome sequencing, but corresponding phenotypic resistance not exhibited.
